# Effects of cooking with liquefied petroleum gas versus biomass on hemoglobin concentrations in pregnant women: a pre-specified exploratory analysis of the HAPIN trial

**DOI:** 10.1038/s41467-026-74114-9

**Published:** 2026-06-10

**Authors:** Sheela S. Sinharoy, Wenlu Ye, Ajay Pillarisetti, Sant-Rayn Pasricha, Lisa M. Thompson, Anaite Diaz-Artiga, Usha Ramakrishnan, Ghislaine Rosa, Maggie L. Clark, Dana Boyd Barr, Vigneswari Aravindalochanan, Kyle Steenland, Shirin Jabbarzadeh, Lindsay J. Underhill, Miles A. Kirby, Amy E. Lovvorn, William Checkley, Jennifer L. Peel, Thomas F. Clasen

**Affiliations:** 1 Hubert Department of Global Health, Rollins School of Public Health, Emory University, Atlanta, GA USA; 2 Division of Environmental Health Sciences, University of California at Berkeley, Berkeley, California, USA; 3 Population Health and Immunity Division, The Walter and Eliza Hall Institute (WEHI), Melbourne, Australia; 4 School of Nursing, University of California San Francisco, San Francisco, CA USA; 5 Center for Health Studies, Universidad del Valle de Guatemala, Guatemala City, Guatemala; 6 Public Health, Policy & Systems, University of Liverpool, Liverpool, United Kingdom; 7 Department of Environmental and Radiological Health Sciences, Colorado State University, Fort Collins, CO USA; 8 Gangarosa Department of Environmental Health, Rollins School of Public Health, Emory University, Atlanta, GA USA; 9 Department of Environmental Health Engineering, Sri Ramachandra Institute of Higher Education and Research, Chennai, Tamil Nadu India; 10 Department of Biostatistics and Bioinformatics, Rollins School of Public Health, Emory University, Atlanta, GA USA; 11 Washington University School of Medicine, Washington University in St Louis, St Louis, MO USA; 12 Department of Global Health and Population, Harvard T.H. Chan School of Public Health, Boston, MA USA; 13 Division of Pulmonary and Critical Care, School of Medicine, Johns Hopkins University, Baltimore, MD USA; 14 Biomedical Research Unit, Asociación Benéfica PRISMA, Lima, Peru; 15 Department of Epidemiology and Biostatistics, College of Public Health, University of Georgia, Athens, GA USA; 16 Department of Environmental Health Science, University of Georgia, Athens, GA USA; 17 Bloomberg School of Public Health, Johns Hopkins University, Baltimore, MD USA; 18 Eagle Research Center, Kigali, Rwanda; 19 Universidad Peruana Cayetano Heredia, Lima, Peru; 20 Nuffield Department of Women’s and Reproductive Health, University of Oxford, Oxford, United Kingdom; 21 Berkeley Air Monitoring Group, Berkeley, California, USA; 22 Department of Epidemiology, Rollins School of Public Health, Emory University, Atlanta, GA USA; 23 Division of International Epidemiology and Population Studies, Fogarty International Center, National Institutes of Health, Bethesda, MD USA; 24 Division of Healthcare Delivery Research, MedStar Health Research Institute, Columbia, MD USA; 25 Division of Developmental Medicine, Boston Children’s Hospital, Harvard Medical School, Boston, MA USA

**Keywords:** Environmental impact, Anaemia, Developing world, Energy access

## Abstract

Evidence linking household air pollution exposure and blood hemoglobin concentration is lacking. We examine the effect of a liquefied petroleum gas cookstove and fuel intervention on hemoglobin concentration, along with associations between household air pollution exposures and hemoglobin concentration, among pregnant women. We enroll 800 pregnant women each in Guatemala, Peru, India, and Rwanda in an open-label randomized controlled trial (NCT02944682). In 3178 women (intervention=1585; control=1593), we measure hemoglobin concentration and 24-hour personal exposure to particulate matter with an aerodynamic diameter ≤2.5μm (PM_2.5_), black carbon (BC), and carbon monoxide (CO) at three timepoints (9-20, 24-28, and 32-36 weeks gestation). We evaluate the effects of the intervention on hemoglobin concentration and conduct exposure-response analyses to examine associations between 24-hour personal exposure to measured pollutants and hemoglobin concentration. We identify a significant increase in hemoglobin in the intervention group (0.074 g/dL, 95% CI: 0.002, 0.145) compared to the control group. In exposure-response analyses, each 1ppm increase in CO exposure is associated with a 0.015 g/dL (95% CI: 0.008, 0.023) increase in hemoglobin. In our analyses, neither PM_2.5_ nor BC are associated with hemoglobin concentration. Further research may be needed to examine the biological mechanisms underlying our findings.

## Abstract (web summary)

Evidence linking household air pollution exposure and blood hemoglobin concentration is lacking. Here, the authors show a positive effect of a liquefied petroleum gas cookstove and fuel intervention on hemoglobin concentrations among pregnant women in Guatemala, Peru, India, and Rwanda.

## Introduction

Household air pollution is a leading risk factor for morbidity and mortality in low- and middle-income countries (LMICs)^1^. The main contributor to household air pollution is cooking with solid fuels (e.g., wood, charcoal, dung, and agricultural residue) with inefficient technologies. About 2.1 billion people worldwide still relied on polluting fuels and technologies for cooking in 2022^2^. Household air pollution generated from these inefficient cooking practices contains a mixture of harmful components, including particulate matter (PM), black carbon (BC), and carbon monoxide (CO). Women typically bear primary responsibility for cooking at the household level and therefore are at the highest risk of exposure to household air pollution from polluting fuels and stoves.

Similarly, women in LMIC settings are at high risk of anemia, defined by the World Health Organization (WHO) as blood concentrations of hemoglobin less than 12 g/dL (or less than 11 g/dL during pregnancy)^3,4^. Among women of reproductive age (15-49 years), global anemia prevalence in 2021 was 33.7% and was higher among pregnant women and in sub-Saharan Africa and Asia^3^. The most common cause of anemia is iron deficiency, which can develop when the body’s iron stores are insufficient to meet individual physiological requirements^5^. Iron deficiency is particularly common during pregnancy, when physiologic iron demand is highest^5^. In LMIC settings, dietary iron intake may be insufficient to meet pregnancy iron requirements; infection and systemic inflammation are also prevalent, further contributing to iron deficiency^5^. As a result, pregnant women in LMICs face multiple risks of iron deficiency and reduced hemoglobin, leading to anemia. Potential adverse outcomes associated with anemia during pregnancy include preterm birth, low birthweight, and postpartum hemorrhage^5^.

While considerable efforts have been made to reduce anemia globally, there has been little progress^3^, and evidence is lacking on whether and how household air pollution may influence hemoglobin concentration and anemia. Household air pollution can lead to inflammation^6^ and may, therefore, contribute to anemia of inflammation, in which systemic inflammation induces hepcidin (a liver-derived hormone that regulates systemic iron levels) and limits body iron supplies^5^. At the same time, CO may have the opposite effect, as it is known to bind with hemoglobin to form carboxyhemoglobin, leading to hypoxia and, hence, increased erythropoiesis and increased hemoglobin production^7^. Reductions in CO exposure may therefore lead to physiologic reductions in hemoglobin and, concurrently, an apparent increase in prevalence of anemia. However, to our knowledge, no studies have examined the effects of a clean household energy intervention on hemoglobin/anemia or the longitudinal association between household air pollution and hemoglobin concentrations or anemia; all existing evidence comes from cross-sectional studies^8–11^.

To address this evidence gap, we leveraged the multicenter, multinational Household Air Pollution Intervention Network (HAPIN) study, a randomized controlled trial (RCT) comparing liquefied petroleum gas (LPG) fuel and cookstove use to ongoing use of biomass fuels and traditional cookstoves by pregnant women. We conducted analyses to determine the effect of the HAPIN intervention on hemoglobin concentration and anemia and to examine associations between exposure to individual pollutants – specifically, particulate matter with an aerodynamic diameter ≤2.5 μm (PM_2.5_), black carbon, and CO – and hemoglobin concentration.

## Results

**Fig. 1:**
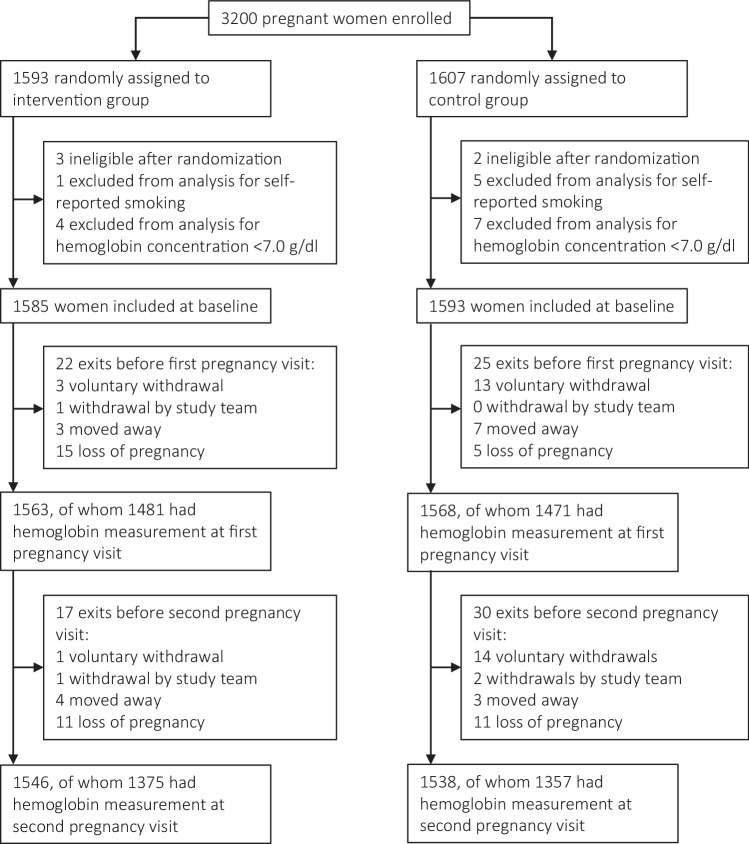
Trial profile.

We randomized 3200 pregnant women from India, Peru, Guatemala and Rwanda between May 7, 2018, and February 29, 2020; five participants were determined ineligible after randomization (Fig. 1). Six participants were excluded from the analysis for current or former smoking, and 11 were excluded for having baseline hemoglobin concentrations <7.0 g/dL at baseline (Fig. 1). The resulting analytic sample consisted of 3178 pregnant women.

### Participant characteristics

Participant characteristics at baseline were similar across the intervention and control groups (Table 1). Participants had a mean age of approximately 25 years and mean gestational age between 15–16 weeks. Approximately one-third of participants had completed secondary school or higher. The mean hemoglobin concentration at baseline was 11.5 g/dL in the control group and 11.4 g/dL in the intervention group (Table 1).

**Table 1:** Household- and individual-level baseline characteristics among the analytic population (*N* = 3178), by study arm

Variable	Control (*N* = 1593)	Intervention (*N *= 1585)
***Household baseline characteristics***		
Household size, mean (SD) [range]	4.3 (2.0) [1–18]	4.3 (2.0) [1–17]
Someone in the household smokes, N (%)		
Yes	178 (11%)	153 (10%)
No	1412 (89%)	1431 (90%)
Missing	3 ( < 1%)	1 ( < 1%)
***Individual (maternal) baseline characteristics***		
Age in years, mean (SD) [range]	25.4 (4.5) [18–35]	25.3 (4.4) [18–35]
Hemoglobin concentration, g/dL, mean (SD) [IQR]	11.5 (1.4) [10.6–12.5]	11.4 (1.5) [10.5–12.4]
Anemia Categories, N (%)		
Severe (Hemoglobin [Hb, g/dL] <7.0)	3 (0.2%)	4 (0.3%)
Moderate (>=7.0, <10.0)	229 (14.4%)	239 (15.1%)
Mild (>=10.0, <11.0)	292 (18.3%)	302 (19.1%)
Mon-Anemia (>=11.0)	1048 (65.8%)	1023 (64.5%)
Missing	21 (1.3%)	17 (1.1%)
Pregnant women with any anemia, N (%)	524 (32.9%)	545 (34.4%)
BMI, kg/m^2^, mean (SD) [range]	23.0 (3.9) [13.7–44.2]	23.3 (4.1) [13.3–42.3]
Underweight ( < 18.5), N (%)	163 (10%)	173 (11%)
Healthy weight ( ≥ 18.5, <25.0), N (%)	966 (61%)	910 (57%)
Overweight ( ≥ 25.0, <30), n (%)	370 (23%)	380 (24%)
Obesity ( ≥ 30.0), n (%)	87 (5%)	110 (7%)
Missing, n (%)	7 (1%)	12 (1%)
Mother’s highest level of education, N (%)*		
No formal education or primary school incomplete	553 (35%)	479 (30%)
Primary school complete or secondary school incomplete	531 (33%)	555 (35%)
Secondary school complete or vocational or some college or university	509 (32%)	550 (35%)
Missing	/	1 ( < 1%)
Gestational age, wk, mean (SD) [range]	15.3 (3.2) [9–24.9]	15.5 (3.1) [9–23.7)
Nulliparous, N (%)*		
Yes	583 (37%)	638 (40%)
No	1008 (63%)	943 (60%)
Missing	2 ( < 1%)	4 ( < 1%)
Household food insecurity score, n (%)*		
Severe/moderate	268 (17%)	218 (14%)
Mild	446 (28%)	415 (26%)
None	857 (54%)	928 (59%)
Missing	22 (1%)	24 (1%)
Mother’s minimum diet diversity, N (%)		
High	165 (10%)	202 (13%)
Medium	527 (33%)	495 (31%)
Low	900 (56%)	887 (56%)
Missing	1 ( < 1%)	1 ( < 1%)

Note: Summary based on 3178 participants included in analysis.

* Significantly different at α = 0.05, based on results of two-sided t test (continuous variables) or non-directional χ2 test (categorical variables) for the difference between intervention and control groups

HAPIN India, Peru, Guatemala, and Rwanda, 2018–2020.

### Exposure measurements

While personal PM_2.5_, BC, and CO exposures were similar at baseline between the intervention and control arms, participants in the intervention arm had significantly lower exposures post-randomization to all three pollutants than those in the control arm. The median [IQR] of valid personal PM_2.5_, BC, and CO exposure measurements by study visit (baseline, follow-up visits 1 and 2) and study arm (intervention vs. control) among the 3178 pregnant women included in the statistical analyses are presented in the Supplement (Table S2). About 70% of the measured post-randomization PM_2.5_ exposures in the intervention arm were below the 2021 WHO annual PM_2.5_ Interim Target 1 of 35 μg/m^3^. The median post-randomization BC and CO exposures in the intervention arm were also reduced from 10.6 μg/m^3^ and 1.32 ppm to 2.8 μg/m^3^ and 0.19 ppm, respectively. Detailed exposure results for these pregnant women participants are described elsewhere^12^.

### Hemoglobin concentrations and anemia

In Table 2, we present the mean, standard deviation (SD), and inter-quartile range (IQR) of hemoglobin concentration and the point prevalence of anemia by study visit (baseline, follow-up visits 1 and 2) and study arm (intervention vs. control) in the pooled sample and in each country site, among the 3178 pregnant women included in the statistical analyses. Mean hemoglobin concentrations differed by study site, with the lowest values for India, and also decreased from baseline to follow-up visit 1 and then increased at follow-up visit 2 in both the intervention and control groups. The average post-randomization hemoglobin concentration was 11.1 g/dL in both control and intervention groups.

**Table 2:** Hemoglobin concentrations (g/dL) and prevalence of anemia at each time point, by study arm, in the pooled sample and each country site

	Control				Intervention			
Time point	*N*	Mean (SD)	IQR	N (%) Anemic	*N*	Mean (SD)	IQR	*N* (%) Anemic
***Pooled – all country sites***								
Baseline	1593	11.5 (1.4)	10.6-12.5	524 (32.9%)	1585	11.4 (1.5)	10.5-12.4	545 (34.4%)
Visit 1	1471	10.9 (1.4)	10.0-12.0	700 (47.6%)	1481	11.0 (1.4)	10.1-12.0	686 (46.3%)
Visit 2	1357	11.3 (1.4)	10.3-12.2	514 (37.9%)	1375	11.2 (1.4)	10.3-12.0	544 (39.6%)
***Guatemala***								
Baseline	400	12.3 (1.0)	11.6-12.9	39 (9.8%)	400	12.1 (1.0)	11.6-12.8	47 (11.8%)
Visit 1	385	11.6 (1.1)	11.0-12.3	92 (23.9%)	392	11.8 (1.0)	11.1-12.4	90 (23.0%)
Visit 2	374	12.0 (1.0)	11.3-12.7	58 (15.5%)	382	11.9 (1.0)	11.2-12.6	73 (19.1%)
***India***								
Baseline	394	10.5 (1.3)	9.6-11.3	255 (64.7%)	396	10.4 (1.2)	9.7-11.2	269 (67.9%)
Visit 1	374	10.1 (1.3)	9.4-11.0	278 (74.3%)	370	10.2 (1.2)	9.4-11.0	268 (72.4%)
Visit 2	336	10.4 (1.4)	9.5-11.5	207 (61.6%)	331	10.5 (1.3)	9.7-11.4	203 (61.3%)
***Peru***								
Baseline	398	11.2 (1.2)	10.4-12.0	149 (37.4%)	396	11.2 (1.3)	10.5-11.9	147 (37.1%)
Visit 1	330	10.5 (1.1)	9.8-11.3	207 (62.7%)	345	10.6 (1.2)	9.9-11.4	208 (60.3%)
Visit 2	308	10.7 (1.2)	9.9-11.4	150 (55.6%)	308	10.7 (1.2)	9.9-11.4	175 (56.8%)
***Rwanda***								
Baseline	401	12.0 (1.5)	11.2-13.0	81 (20.2%)	393	12.0 (1.6)	11.2-13.1	82 (20.9%)
Visit 1	382	11.4 (1.6)	10.6-12.3	123 (32.2%)	374	11.4 (1.5)	10.5-12.5	120 (32.1%)
Visit 2	377	11.7 (1.4)	10.9-12.5	99 (26.3%)	354	11.7 (1.5)	10.9-12.7	93 (26.3%)

HAPIN India, Peru, Guatemala, and Rwanda, 2018–2020.

### ITT analysis

In Table 3, we present the results of the ITT analysis using a mixed-effects model that compares the post-randomization hemoglobin concentration during pregnancy by study arm. Trial-wide, hemoglobin in the intervention group was 0.074 g/dL (degrees of freedom = 2902, 95% CI: 0.002, 0.145) higher than that in the control group. Country-specific ITT analyses showed a similar trend, with the largest increase in India (0.141 g/dL), though none of the country-specific intervention effects reached statistical significance at *p* < 0.05 (Table 3). In analyses of effects on anemia as a binary variable, in trial-wide and country-specific analyses, women in the intervention group had similar odds of anemia as those in the control group (Table 3).

**Table 3:** ITT analysis results from mixed-effects models

Hemoglobin concentration (continuous)^a^	N	Estimate (g/dL)	95% CI	*p*-value
**Pooled (all participants)**	2972	0.074	(0.002, 0.145)	0.042
**Guatemala**	778	0.076	(−0.041, 0.193)	0.205
**India**	768	0.141	(−0.006, 0.288)	0.061
**Peru**	660	0.016	(−0.129, 0.162)	0.826
**Rwanda**	766	0.039	(−0.119, 0.196)	0.631
**Anemia (binary)**^**b**^	**N**	**Odds Ratio**	**95% CI**	***p***-**value**
**Pooled (all participants)**	2972	0.948	(0.814, 1.103)	0.489
**Guatemala**	778	0.989	(0.722, 1.354)	0.943
**India**	768	0.893	(0.665, 1.201)	0.455
**Peru**	660	0.951	(0.713, 1.268)	0.731
**Rwanda**	766	0.989	(0.716, 1.366)	0.945

**Note:** Models controlled for baseline hemoglobin level and randomization strata.

Linear mixed-effects model was used for continuous hemoglobin outcome, and logistic mixed-effects model was used for anemia outcome.

^a^Estimates were obtained from a linear mixed-effects model (random intercept for household ID). Two-sided t tests were used. No adjustment was made for multiple comparisons.

^b^Estimates were obtained from a logistic mixed-effects model (random intercept for household ID). Two-sided Wald tests were used. No adjustment was made for multiple comparisons.

HAPIN India, Peru, Guatemala, and Rwanda, 2018-2020.

### Exposure-response analysis

Results for the adjusted associations between PM_2.5_, black carbon, and CO exposure and adjusted hemoglobin concentrations, trial-wide and by country site, are shown in Table 4. Evaluation of different models indicated that the log-linear model fit better for PM_2.5_ and black carbon, and the linear model fit better for CO. We did not observe a clear pattern for non-linear exposure-response relationships. Results for these best-fitting models are presented in Table 4. Full results (in linear, log-linear, and categorical forms) of unadjusted and adjusted exposure-response models for each time point and for the mixed-effects models are presented in the Supplement.

**Table 4:** Parameter estimates from exposure-response analysis using mixed-effects regression models of the association of each pollutant (PM_2.5_, BC, or CO) with hemoglobin concentration – pooled and by country site

Exposures	Model Type	Estimate (g/dL)	95% CI	*p*-value
***Pooled – all country sites***				
PM_2.5_	Log linear	−0.001	(−0.032, 0.030)	0.953
Black carbon	Log linear	0.015	(−0.018, 0.049)	0.372
CO	Linear	0.015	(0.008, 0.023)	<0.001
***Guatemala***				
PM_2.5_	Log linear	0.005	(−0.042, 0.052)	0.831
Black carbon	Log linear	−0.012	(−0.079, 0.055)	0.722
CO	Linear	−0.007	(−0.024, 0.011)	0.460
***India***				
PM_2.5_	Log linear	-0.005	(−0.069, 0.058)	0.869
Black carbon	Log linear	0.014	(−0.043, 0.071)	0.638
CO	Linear	0.010	(−0.007, 0.028)	0.258
***Peru***				
PM_2.5_	Log linear	0.027	(−0.031, 0.084)	0.361
Black carbon	Log linear	0.044	(−0.014, 0.10)	0.133
CO	Linear	0.016	(0.006, 0.027)	0.002
***Rwanda***				
PM_2.5_	Log linear	−0.044	(−0.128, 0.039)	0.299
Black carbon	Log linear	−0.032	(−0.136, 0.072)	0.542
CO	Linear	0.023	(0.005, 0.040)	0.010

**Note:** All adjusted exposure-response models controlled for maternal age; mother’s highest education level; BMI at baseline; household food insecurity, mother’s diet diversity, and exposure to secondhand smoke at baseline; gestational age and gestational age squared at the hemoglobin measurement; and country.

HAPIN India, Peru, Guatemala, and Rwanda, 2018-2020.

We observed a statistically significant association between CO exposure and hemoglobin concentration, but not between PM_2.5_ or black carbon and hemoglobin concentration, among pregnant women (Table 4). Trial-wide, in the adjusted linear model, a 1-ppm increase in exposure to CO was associated with a positive change in hemoglobin concentration of 0.015 g/dL (95% CI: 0.008, 0.023) g/dL. When scaled to the IQR increase in CO exposure, this corresponds to an estimated increase of 0.027 g/dL (95% CI: 0.014, 0.042) g/dL. As shown in Table 4, country-specific CO-hemoglobin associations generally reflect the trial-wide findings (except for Guatemala), with higher CO exposures associated with higher hemoglobin. The strongest association with hemoglobin was observed in Rwanda, followed by Peru and India. Generally, hemoglobin concentrations were lower with increased exposure to PM_2.5_ but higher with increased exposure to black carbon, although none of these associations reached statistical significance ($\alpha$=0.05). Trial-wide, 1-log-µg/m^3^ increase in PM_2.5_ and BC exposures was associated with -0.001 (95% CI: (-0.032, 0.030) g/dL and 0.015 (95% CI: (-0.018, 0.049) g/dL change in hemoglobin concentration, respectively. When scaled to IQR increase in log-transformed exposures, these correspond to changes of -0.001 g/dL (95% CI: -0.048, 0.045) for PM_2.5_ and 0.022 g/dL (95% CI: -0.027, 0.073) for BC. The PM_2.5_-hemoglobin and black carbon-hemoglobin associations were neither consistent in direction nor significant across country sites.

The exploratory analysis of the effect of the intervention on pregnant women who were anemic at baseline included data from 1009 women. Among these women, hemoglobin in the intervention group was 0.091 g/dL (95% CI: −0.044, 0.225) higher than that in the control group. The increase was largest in India (0.155 g/dL, 95% CI: −0.029, 0.339), though none of the results reached statistical significance at *p* < 0.05. Full results are presented in the Supplement (Table S23). In the exploratory analysis based on timing of study enrollment, hemoglobin in the early enrollment intervention group was 0.099 g/dL (95% CI: −0.007, 0.205) higher than that in the control group, while hemoglobin in the late enrollment intervention group was 0.044 g/dL (95% CI: -0.051, 0.139) higher than that in the control group. However, these results did not reach statistical significance at *p* < 0.05.

## Discussion

In this individually randomized controlled trial conducted across four country contexts, we observed a positive effect of an LPG cook stove and free fuel intervention on hemoglobin concentrations among pregnant women. The effect on hemoglobin concentration was strongest in India, the study site with the lowest baseline hemoglobin concentrations. The effect size, while statistically significant, was small and likely not clinically meaningful at the individual level. The odds of anemia did not differ between women in the intervention and control groups, likely because the small effect on hemoglobin concentration was insufficient to change anemia status. In exposure-response analyses, we observed that higher personal exposure to CO was significantly associated with higher hemoglobin concentrations trial-wide and in the Peru and Rwanda sub-samples. We did not observe any significant associations between personal exposures to particulate matter (as measured by PM_2.5_ or black carbon) and hemoglobin. To our knowledge, our study is the first intervention study to evaluate the effects of a cleaner cookstove intervention on hemoglobin concentrations and, as such, provides the most rigorous evidence to date on relationships between household air pollution and hemoglobin concentrations among pregnant women.

The effect size observed in our study (0.074 g/dL) is small, especially compared to interventions designed to increase iron status through direct approaches for an individual person or patient. For example, the standard of care for pregnant women is daily oral iron and folic acid (IFA) supplementation^13^. A recent systematic review and meta-analysis found that daily oral iron supplementation had a mean effect size on hemoglobin concentration among pregnant women at or near term ( ≥ 34 weeks of gestation) of 0.953 g/dL (95% CI: 0.699, 1.206)^14^. RCTs of other interventions to increase iron intake among pregnant women through dietary supplementation have had mixed results: iron-fortified beverages were found to increase hemoglobin concentrations (0.416 g/dL) in Tanzania but not in Vietnam, while daily food supplementation was found to have no effect on hemoglobin concentration in Indonesia^15–17^. Another RCT of iron-fortified food supplementation among pregnant women in Cambodia did not report hemoglobin concentrations but reported a significant protective effect on anemia (Odds Ratio = 0.51; 95% CI: 0.34, 0.77)^18^. The observed effect size in our trial, while not clinically meaningful alone, is larger than has been observed in some dietary interventions and could complement the standard of care IFA supplementation as part of a larger public health, population-level anemia prevention and control strategy.

The biological mechanism underlying the intervention's observed positive effect on hemoglobin concentrations remains unclear. The exposure-response results, which indicated no association between PM_2.5_ or BC and hemoglobin concentration, did not provide evidence in support of a pathway through reduced air pollution. However, household air pollution that results from cooking with biomass fuel is known to contain several other inflammatory agents that were unmeasured in our study^19,20^. The LPG stove and free fuel intervention resulted in large reductions in personal exposures to measured pollutants^12^, suggesting that a causal pathway from the intervention to increased hemoglobin concentrations through reduced inflammation remains biologically plausible. There may also be other pathways unrelated to inflammation that could have been impacted by the intervention, such as through diet. Further research is needed to examine exposure to pollutants, biomarkers of inflammation, and hemoglobin concentration, to identify biological pathways of effect.

The exposure-response results for the positive association between CO and hemoglobin concentration are biologically plausible and are likely due to the well-known relationship between tissue hypoxia and hemoglobin, in which a diminished availability of oxygen can lead to secondary, or compensatory, erythrocytosis^21^. Several cross-sectional studies have observed significantly higher carboxyhemoglobin and hemoglobin concentrations in cigarette smokers compared to non-smokers, an association that is hypothesized to be due to smokers’ higher CO exposure^21–24^. Exposure to household air pollution may have a similar physiological effect to cigarette smoking, in which direct inhalation of CO increases hemoglobin concentration. In our study, since hemoglobin was often measured after a morning cooking event, our data may capture a process in which CO inhalation leads to a corresponding physiologic increase in hemoglobin concentration.

Our findings related to CO also align with studies from rural areas of Guatemala and India, which documented evidence of chronic low-level CO exposures linked to household air pollution from cooking with solid biomass^8,25,26^. Of these prior studies, two measured CO exposure through exhaled breath CO levels^8,26^, while one measured CO in indoor air using gas-solid chromatography and found concentrations consistently greater than 10 ppm, substantially higher than those in our study^25^. Another study from rural India found high carboxyhemoglobin levels among women regardless of cooking fuel type, suggesting exposure to CO from sources other than cookstoves^27^. CO levels in our study were low at baseline, limiting the potential for further reductions and for stronger exposure-response effects.

Among previous studies examining household air pollution and hemoglobin concentrations, all were cross-sectional, limiting causal inference. Only one study targeted pregnant women, examining associations between cooking fuel type and hemoglobin concentration in a cohort study in India; results indicated a positive association between use of biomass cooking fuels (compared to clean fuels such as LPG and electricity) and anemia^11^. Other observational studies in Guatemala, Sri Lanka, and India focused on non-pregnant women of reproductive age and produced mixed results. The first study enrolled 274 women in rural Guatemala who cooked with biomass fuels; results indicated no difference in hemoglobin concentrations between those who used a smokeless stove with a chimney and those who cooked over smoky open fires without functioning chimneys^8^. The second enrolled 382 women in Sri Lanka and similarly found no association between hemoglobin concentration and type of cooking fuel (firewood, LPG, or kerosene)^9^. A third study of 60 women in India observed that hemoglobin concentration was inversely associated both with wood fuel use (compared to LPG) and measured levels of PM_2.5_^10^. Our study builds on this previous research in several ways, including leveraging a larger study population, a more rigorous study design that allows for causal attribution, and a higher-quality LPG cookstove that effectively reduced exposure to key pollutants.

Additional strengths of our study are the inclusion of diverse settings across our multi-country trial, which increases generalizability of the results; that the intervention was delivered and adopted with high fidelity; and that PM_2.5_, black carbon, and CO exposures were significantly and consistently lower in the intervention arm than in the control arm^12,28^. Limitations include the use of the HemoCue point-of-care device, which has clinically acceptable performance but is less precise than the gold standard method (i.e., laboratory-based hematology analyzer with venous blood)^29^. Future studies using venous blood and that measure biomarkers of micronutrient status and inflammation, including in other populations at high risk of anemia, such as young children and non-pregnant women, may be useful. Future analyses may also examine the effect of cleaner cooking interventions on maternal morbidity and adverse birth outcomes by anemia category; such analyses were outside the scope of our current study. Our main ITT result, indicating an effect size of 0.074g/dL, had a 95% confidence interval of 0.002 to 0.145g/dL, suggesting some uncertainty in the estimate. Finally, although our exposure-response analysis accounted for several covariates, there may still be unmeasured or residual confounding. For example, HAPIN did not collect data on the consumption of IFA supplements, which could have influenced hemoglobin concentration. However, because of the RCT design, we anticipate that any unmeasured covariates will be balanced between the intervention and control arms.

The effect of the LPG stove and free fuel intervention on hemoglobin concentration indicates a potential role for the use of cleaner cookstoves to improve hemoglobin levels in women of reproductive age at the population level. Currently, anemia prevention and reduction strategies focus on increasing iron intake, because dietary iron deficiency remains the primary cause of anemia worldwide^3^. The WHO framework for action on anemia reduction includes mentions of environmental factors but focuses only on water, sanitation, and hygiene, and does not include any mention of household air pollution, likely because of previously limited evidence for causal relationships^30,31^. However, our evidence suggests that cleaner cooking programs and policies to reduce both household and ambient air pollution could support multisectoral collaborative efforts to prevent and reduce anemia by addressing its complex, multifactorial causes.

## Methods

### Study design and participants

We conducted an RCT of LPG cookstoves and free fuel distribution compared to traditional biomass burning stoves. We identified pregnant women through antenatal clinics in ten geographic strata across four countries: one district in Jalapa, Guatemala; two districts in Tamil Nadu, India; six provinces in Puno, Peru; and one district in Eastern Province, Rwanda. Inclusion criteria were being 18-34 years old and pregnant with a viable singleton fetus of 9-20 weeks gestation confirmed by ultrasound. Exclusion criteria were current tobacco smoking, living outside of the trial area (or planning to move permanently outside of the trial area within 12 months), or cooking primarily with LPG or another clean fuel (e.g., electric) or likely to start doing so during the study. Details of the study design and methodology are described elsewhere^32–34^.

### Ethics and inclusion

The study protocol was reviewed and approved by institutional review boards or ethics committees at Emory University (00089799), Johns Hopkins University (00007403), Sri Ramachandra Institute of Higher Education and Research (IEC-N1/16/JUL/54/49), the Indian Council of Medical Research – Health Ministry Screening Committee (5/8/4-30/(Env)/Indo-US/2016-NCD-I), Universidad del Valle de Guatemala (146-08-2016/11-2016), Guatemalan Ministry of Health National Ethics Committee (11-2016), A.B. PRISMA (CE2981.17; CE2008.18; CE0028.20; CE0291.21), the London School of Hygiene and Tropical Medicine (11664-5), the Rwandan National Ethics Committee (No.853/RNEC/2016; 317/2017; 357/2018; 194/2019; 929/2020; 64/2021), and Washington University in St. Louis (201611159). Participants provided written informed consent. The trial was registered on clinicaltrials.gov (Identifier NCT02944682), where the study protocol is also available.

The study involved close collaboration with local researchers at all four study sites throughout the research process. Roles and responsibilities were discussed with local research teams and clearly outlined in advance. Local collaborators played an integral role in the design of both an initial formative study and the main trial. They participated in regular meetings to ensure the research was locally relevant and contributed to developing study procedures and survey questions tailored to each site's specific context. To foster capacity building, study teams implementing the research were recruited from local communities. Trainings were developed with a strong emphasis on strengthening local research capacities, particularly in areas such as research ethics, survey methods, specimen collection, laboratory procedures, and data management. Remuneration for control participants was carried out in accordance with local ethics committee guidelines and cultural suitability. The research did not result in any stigmatization, incrimination, discrimination, or other personal risk to participants. In response to the COVID-19 pandemic, in-person data collection was paused to protect both the research teams and participants from potential exposure. Several local collaborators contributed as co-authors to the current manuscript, while additional collaborators are recognized as HAPIN Investigators and are listed in the Acknowledgements section. Finally, local and regional research relevant to our study is included in the citations of this manuscript.

### Randomization and masking

Participants were randomly assigned in a 1:1 ratio stratified by setting (ten geographic strata, as listed above) in permuted blocks of two and four to either receive the intervention or continue their traditional cooking practices with biomass fuels. We sought to randomly assign 1600 participants to the intervention arm and 1600 to the control arm. Only one pregnant woman per household was allowed to participate. The HAPIN Data Management Core generated randomization lists based on block randomization using randomly selected block sizes, then prepared individual sealed envelopes containing trial group allocation, which were shipped to each study site. Field teams then followed a detailed protocol in which each household was presented with a set of six sealed, sequenced envelopes and asked to select one, which contained their study arm assignment. Due to the nature of the intervention, it was not possible to mask participants or data collection teams from the group assignment. However, the study investigators were blinded to the collected data, and primary analyses were conducted on blinded data. Participants in the control group received compensation, which varied by country, to offset the economic benefit of the intervention^35^.

### Procedures and outcomes

We enrolled 800 women per country and individually randomized them to intervention or control groups, with intervention households receiving an LPG stove, continuous free fuel delivery, and behavioral reinforcements for 18 months^36^. Women and their infants were followed through the first year of the infant’s life. The sample size was determined based on the primary outcomes of the trial, which were low birth weight, severe pneumonia, and stunting in infants, along with high blood pressure in non-pregnant adult women residing in the household^32^. Results related to the first three of these primary outcomes have been published elsewhere; results related to the fourth primary outcome are forthcoming^37–39^. Secondary outcomes included maternal blood pressure, fetal growth, and infant development; results for these outcomes have been published elsewhere^40–42^. A full list of pre-planned secondary outcomes and their publication status is provided in the Supplement (Table S1). Hemoglobin concentration and anemia are identified in the study protocol and trial registration as other outcomes.

Data were collected in person at the household level by trained staff. Data on socio-demographic characteristics, medical history, and cooking and other behavioral practices were collected from pregnant women at baseline (9-20 weeks gestation) using structured survey modules that were programmed on tablets equipped with the Research Electronic Data Capture (REDCap) mobile application^43,44^. Hemoglobin concentration was measured from a single drop of capillary blood obtained via finger prick, using the HemoCue® Hb 201+ System, during household visits at baseline and then at two follow-up time points (24-28 and 32-36 weeks of gestation). These time points were selected based on logistical feasibility for the field teams. Field technicians were trained (and re-trained at yearly intervals) to follow standardized procedures for finger-stick capillary blood sampling. Details of data collection procedures for demographics and hemoglobin concentration among pregnant women are described elsewhere^32,33^.

We measured personal exposure to PM_2.5_, black carbon, and CO in pregnant women at the same three time points described above^34^. These three pollutants were selected because they are known to be products of incomplete household fuel combustion and because of their associations with adverse health outcomes^34^. We used the Enhanced Children’s MicroPEM™ (ECM) (RTI International), a lightweight and validated personal monitor to assess 24-hour exposure to PM_2.5_. The ECM is a combined nephelometric and gravimetric sampler with an air flow pump set to operate continuously at 0.3 L/min for 24 h. The ECM collects PM_2.5_ on a filter by drawing air through a size-selective impactor attached to a cassette containing 15-mm Teflon® filters (PT15-AN-PF02; MTL Corporation) and measures real-time PM_2.5_ with a nephelometer^34^. Black carbon on the PM_2.5_ filters was estimated using a SootScan™ Model OT21 transmissometer (Magee Scientific). The instrument measures the light attenuation through the filter, which is then converted into black carbon surface deposition^12,45^. Lascar EL-USB-300 monitors (Lascar Electronics) were used to measure 24-h real-time CO exposures. The Lascar monitor has a sensing range between 0 and 300 ppm and logs CO concentrations at 1-minute intervals.

Participants wore ECM and Lascar monitors in a pocket of a customized garment during the monitoring period. They were instructed to keep the instrumentation nearby (within 1-2 m) when sleeping or conducting activities that may damage the equipment. 24-hour average personal exposures to PM_2.5_ (based on gravimetric samples), black carbon, and CO were used in exposure-response analysis. If a gravimetric PM_2.5_ sample was considered invalid, measurement-specific nephelometric concentration was used instead^12^. Detailed sampling instrumentation, sampling strategy and quality control and assurance are described elsewhere^12,34^.

### Statistical analysis

Data presented in this manuscript were analyzed in accordance with an analysis plan that was pre-specified and approved prior to securing data access. Participants were excluded from the statistical analysis if they reported any current smoking, in line with trial exclusion criteria. Participants with a hemoglobin concentration <7.0 g/dL at baseline were referred for additional management of their anemia. These participants were also dropped from our analysis as we anticipated that their anemia management would affect our outcome of interest. We adjusted hemoglobin concentrations for altitudes ≥1000 meters using a standard formula^46^. Elevation data were collected at the household level in Guatemala and Rwanda using the GPS feature within the REDCap application. In Peru, elevation data were unavailable, so we used Puno's elevation (3,827 meters) for all households. Adjustment was not necessary for India, as all households were <1000 meters.

We conducted an intention-to-treat (ITT) analysis to assess the effect of the LPG cookstove and fuel intervention on hemoglobin in pregnant women using a mixed-effects model. The two post-randomization hemoglobin measurements were regressed on study arm, with an indicator variable for the ten randomization strata in which randomization occurred (as described above) and a random intercept for each individual, controlling for baseline/pre-randomization hemoglobin levels.

We created a binary variable for anemia using the WHO cut-off for pregnant women, in which anemia is defined as an adjusted hemoglobin concentration of less than 11 g/dL^4^. We then conducted an ITT analysis to assess the effect of the LPG cookstove and fuel intervention on binary anemia status, using logistic regression with random effects.

We also conducted exposure-response analyses to assess the associations between personal exposure to each pollutant (PM_2.5_, black carbon, and CO) and adjusted hemoglobin concentrations. Again, we used mixed-effects models and regressed the three hemoglobin measurements on the three exposure measurements assessed during the same visit. Given the moderate to high correlations between the exposures to the three pollutants and their different potential impacts on hemoglobin, we modeled the associations for PM_2.5_, black carbon, and CO separately^12^. In all exposure-response analyses, we included a random intercept for each participant, time-varying gestational age and gestational age squared at each visit (because hemoglobin decreases and then rises during pregnancy), and other time-invariant covariates.

A priori covariate selection for exposure-response models was guided by a directed acyclic graph (Supplemental Fig. S1) that was developed based on previous literature^5,47^. We identified potential confounders as any variables reported in the literature to be associated with both the exposure and the outcome and not on the causal path nor a collider between the exposure and the outcome. In addition to confounders, we included variables in the models if they were hypothesized to be predictors of the outcome, as this is known to decrease the amount of unexplained variability in the data and increase power to estimate an association^48^. The time-varying covariates were gestational age and gestational age squared (as hemoglobin level is known to decrease then rise during pregnancy, Fig. S2) at each visit^49^. The time-invariant covariates were maternal age, mother’s highest education completed, mother’s body mass index at baseline, household food insecurity, mother’s diet diversity, exposure to secondhand smoke, and country site.

For each exposure-response relationship, we first fit linear models with different exposure forms (i.e., linear and log-linear). Log-linear models are commonly used in air pollution epidemiology to better capture relative exposure-response relationships, improve model fit, and reduce the influence of extreme exposure values. We also evaluated potential nonlinear patterns by fitting a categorical (quartile) exposure model. We assessed model fit using Akaike’s Information Criterion (AIC); results for best-fitting models are presented above, while results for other models are presented in the Supplement. We also conducted country-specific ITT and exposure-response analyses using the same approach as described above. As secondary exposure-response analyses, we examined the associations between PM_2.5_, black carbon, and CO and adjusted hemoglobin separately at each visit.

We conducted two additional exploratory analyses. First, we evaluated the effect of the LPG cookstove and fuel intervention on hemoglobin in the sub-group of pregnant women who were anemic at baseline, using the same analytic approach as for the main ITT analysis. Second, we evaluated the effect of the intervention according to gestational age at study enrollment, with early enrollment being defined as baseline data collection occurring before 15 weeks gestation. In this second analysis, the early and late enrollment groups were each compared with the control group. Further details on statistical analyses are provided in an Extended Methods section in the Supplemental Materials.

Missing or invalid exposure and hemoglobin measurements were dropped from the model. A *p*-value of <0.05 was considered statistically significant. All tests were two-tailed and, in line with published guidance, we did not adjust for multiple comparisons^50,51^. All analyses were performed using R version 4.2.2. For statistical modeling, we used the R package ImerTest version [1] ‘3.1.3’.

### Role of the funding source

The sponsors did not have a role in study design; in the collection, analysis, and interpretation of data; in the writing of the report; or in the decision to submit this paper for publication.

### Reporting summary

Further information on research design is available in the Nature Portfolio Reporting Summary linked to this article.

## Supplementary information

Supplementary Information

Reporting summary

Transparent Peer Review file

## Source data

Source data

## Data Availability

The data supporting the findings from this study are available within the manuscript and its supplementary information. The individual de-identified data generated in this study have been deposited in the Emory Dataverse under accession code 10.15139/S3/M4N5QO [10.15139/S3/M4N5QO]. Source data are also available with this paper. Source data are provided with this paper.
