# Supplementary material for: Effects of cooking with liquefied petroleum gas versus biomass on hemoglobin concentrations in pregnant women: a pre-specified exploratory analysis of the HAPIN trial
Source: Nat Commun. 2026 Jun 10;17:7393. doi: 10.1038/s41467-026-74114-9 (PMC13402313; doi:10.1038/s41467-026-74114-9)
Supplement: Supplementary file 1 — Supplementary Information [file 41467_2026_74114_MOESM1_ESM.pdf]

Effects of cooking with liquefied petroleum gas versus biomass on hemoglobin concentrations in pregnant women: a pre-specified exploratory analysis of the HAPIN trial

Supplemental Materials

Sheela S. Sinharoy[1]†, Wenlu Ye[2]†, Ajay Pillarisetti[2], Sant-Rayn Pasricha[3], Lisa M. Thompson[4], Anaité Diaz-Artiga[5], Usha Ramakrishnan[1], Ghislaine Rosa[6], Maggie L. Clark[7], Dana Boyd Barr[8], Vigneswari Aravindalochanan[9], Kyle Steenland[8], Shirin Jabbarzadeh[10], Lindsay Underhill[11], Miles A. Kirby[12], Amy Lovvorn[8], William Checkley[13], Jennifer Peel[7], Thomas F. Clasen[8], and HAPIN investigators

† Authors contributed equally to the work

[1] Hubert Department of Global Health, Rollins School of Public Health, Emory University, Atlanta, Georgia, United States of America
[2] Department of Environmental Health Sciences, University of California at Berkeley, Berkeley, California, United States of America
[3] Population Health and Immunity Division, The Walter and Eliza Hall Institute (WEHI), Melbourne, Australia
[4] Nell Hodgson Woodruff School of Nursing, Emory University, Atlanta, Georgia, United States of America
[5] Center for Health Studies, Universidad del Valle de Guatemala, Guatemala City, Guatemala
[6] Public Health, Policy & Systems, University of Liverpool, Liverpool, United Kingdom
[7] Department of Environmental and Radiological Health Sciences, Colorado State University, Fort Collins, Colorado, United States of America
[8] Gangarosa Department of Environmental Health, Rollins School of Public Health, Emory University, Atlanta, Georgia, United States of America
[9] Department of Environmental Health Engineering, Sri Ramachandra Institute of Higher Education and Research, Chennai, Tamil Nadu, India
[10] Department of Biostatistics and Bioinformatics, Rollins School of Public Health, Emory University, Atlanta, Georgia, United States of America
[11] Washington University School of Medicine, Washington University in St Louis, St Louis, Missouri, United States of America
[12] Department of Global Health and Population, Harvard T.H. Chan School of Public Health, Boston, Massachusetts, United States of America
[13] Division of Pulmonary and Critical Care, School of Medicine, Johns Hopkins University, Baltimore, Maryland, United States of America

Table of Contents

**Extended Methods**	3
**Table S1.** HAPIN secondary outcomes and status of publications	4
**Figure S1.** Directed acyclic graph of hypothesized relationships between household air pollution and hemoglobin concentration	6
**Figure S2**. Adjusted hemoglobin level by gestational age at visit (in days) between the control and intervention groups.	6
**Table S2.** Summary of 24-hour personal $PM_{2.5}$, BC, and CO exposures by study arm and visit	7
**Table S3.** Exposure-response analysis results at baseline – all IRCs	8
**Table S4.** Exposure-response analysis results at baseline – Guatemala	9
**Table S5.** Exposure-response analysis results at baseline – India	10
**Table S6.** Exposure-response analysis results at baseline – Peru	11
**Table S7.** Exposure-response analysis results at baseline – Rwanda	12
**Table S8.** Exposure-response analysis results at post-randomization follow up visit 1 – all IRCs	13
**Table S9.** Exposure-response analysis results at post-randomization follow up visit 1 – Guatemala	14
**Table S10.** Exposure-response analysis results at post-randomization follow up visit 1 – India	15
**Table S11.** Exposure-response analysis results at post-randomization follow up visit 1 – Peru	16
**Table S12.** Exposure-response analysis results at post-randomization follow up visit 1 – Rwanda	17
**Table S13.** Exposure-response analysis results at post-randomization follow up visit 2 – all IRCs	18
**Table S14.** Exposure-response analysis results at post-randomization follow up visit 2 – Guatemala	19
**Table S15.** Exposure-response analysis results at post-randomization follow up visit 2 – India	20
**Table S16.** Exposure-response analysis results at post-randomization follow up visit 2 – Peru	21
**Table S17.** Exposure-response analysis results at post-randomization follow up visit 2 – Rwanda	22
**Table S18.** Exposure-response analysis results from mixed-effects models – all IRCs	23
**Table S19**. Exposure-response analysis results from mixed-effects models – Guatemala	24
**Table S20**. Exposure-response analysis results from mixed-effects models – India	25
**Table S21**. Exposure-response analysis results from mixed-effects models – Peru	26
**Table S22**. Exposure-response analysis results from mixed-effects models – Rwanda	27
**Table S23.** Exploratory analysis results of ITT effects among women who were anemic at baseline – all IRCs and by country	28

Extended Methods

The model for the intention-to-treat analysis of intervention effect on hemoglobin concentration is:

$$Hb_{ij} = \beta_0 + b_i + \beta_1 Arm_i + \beta_2 baseline\_Hb_i + \beta_3 X_{1i} \ldots + \beta_{12} X_{10i} + \varepsilon_{ij}$$

where Hb_{ij} is the hemoglobin level for participant i at visit j (post-intervention follow-up 1 or 2); β_0 is the population intercept; b_i is the individual random intercept; Arm_i is the indicator variable for treatment arm (0 = control and 1 = intervention); $baseline\_Hb_i$ is the pre-intervention hemoglobin level, X_{1i} through X_{10i} are indicator variables for the ten randomization strata, and ε_{ij} is the model residual, assumed to be normally distributed. Coefficient β_1 captures the effect of study arm on post-intervention hemoglobin levels in pregnant women over the gestational period under observation.

The model for the intention-to-treat analysis of intervention effect on anemia (binary) is:

$$logit\big(P(y_{ij} = 1)\big) = \beta_0 + b_i + \beta_1 Arm_i + \beta_2 baseline\_Hb_i + \beta_3 X_{1i} \ldots + \beta_{12} X_{10i}$$

where y_{ij} is the binary anemia status for participant i at visit j (post-intervention follow-up 1 or 2); all other parameters and variables are the same as described above.

The general form of the exposure-response models is:

$$Hb_{ij} = \beta_0 + b_i + \beta_1 Exposure_{ij} + \sum \boldsymbol{\beta Z_{ij}} + \sum \boldsymbol{\beta Z_i} + \varepsilon_{ij}$$

where Hb_{ij} is the hemoglobin level for participant i at visit j (baseline, post-intervention follow-up 1 or 2); β_0 is the population intercept; b_i is the individual random intercept; $Exposure_{ij}$ is personal exposure to $PM_{2.5}$, black carbon, or CO for participant i at visit j, $\boldsymbol{Z_{ij}}$ are time-varying covariates; $\boldsymbol{Z_i}$ are time-invariant covariates; and ε_{ij} is the model residual, assumed to be normally distributed. β_1 is the exposure coefficient of interest, indicating the change in hemoglobin concentrations per unit increase in personal exposures.

Table S1. HAPIN secondary outcomes and status of publications

Secondary Outcome	Published?<br>If yes: Citation
Maternal blood pressure	**Yes**<br>Ye W, Steenland K, Quinn A, et al. Effects of a Liquefied Petroleum Gas Stove Intervention on Gestational Blood Pressure: Intention-to-Treat and Exposure-Response Findings from the HAPIN Trial. Hypertension. 2022;79 (8): 1887–9
Preterm birth	**Yes; included as secondary analyses in primary outcome paper** Clasen TF, Chang HH, Thompson LM, Kirby MA, et al. Liquefied petroleum gas or biomass for cooking and effects on birth weight. New England Journal of Medicine. 2022 Nov 10;387(19):1735-46.
Fetal growth	**Yes**<br>Checkley W, Thompson LM, Hossen S, et al. 2024. Cooking with Liquefied Petroleum Gas or Biomass and Fetal Growth Outcomes: A Multi-Country Randomised Controlled Trial. The Lancet Global Health. 2024;12 (5): e815–25.
Gestational age at birth	**Yes; included as secondary analyses in primary outcome paper**<br>Clasen TF, Chang HH, Thompson LM, et al. Liquefied petroleum gas or biomass for cooking and effects on birth weight. New England Journal of Medicine. 2022 Nov 10;387(19):1735-46.
Infant growth (as a continuous outcome)	**Yes; included as secondary analyses in primary outcome paper**<br>Checkley W, Thompson LM, Sinharoy SS, et al. "Effects of Cooking with Liquefied Petroleum Gas or Biomass on Stunting in Infants." New England Journal of Medicine. 2024 Jan 4;390(1):44-54.
Infant development<br>(CREDI)	**No**<br>In preparation; expected submission June 2026
-WHO IMCI severe pneumonia among children <12 months old<br>*-WHO IMCI non-severe pneumonia*<br>*-WHO Pocketbook pneumonia*<br>*-WHO Pocketbook severe*<br>*-Hypoxemia and/or imaging-confirmed pneumonia*<br>*-Hospitalization for respiratory illness*	**Yes; included as secondary analyses in primary outcome paper**<br>McCollum ED, McCracken JP, Kirby MA, et al. "Liquefied petroleum gas or biomass cooking and severe infant pneumonia." New England Journal of Medicine. 2024 Jan 4;390(1):32-43.
Burns	**Yes; presented as adverse events in primary outcome papers**<br>Clasen TF, Chang HH, Thompson LM, et al. Liquefied petroleum gas or biomass for cooking and effects on birth weight. New England Journal of Medicine. 2022 Nov 10;387(19):1735-46.<br><br>Checkley W, Thompson LM, Sinharoy SS, et al. Effects of Cooking with Liquefied Petroleum Gas or Biomass on Stunting in Infants. New England Journal of Medicine. 2024 Jan 4;390(1):44-54.<br><br>McCollum ED, McCracken JP, Kirby MA, et al. Liquefied petroleum gas or biomass cooking and severe infant pneumonia. New England Journal of Medicine. 2024 Jan 4;390(1):32-43.
Diastolic blood pressure, pulse pressure, mean arterial pressure	**No**<br>Under review; included as secondary analysis in primary systolic blood pressure outcome manuscript: "Liquefied Petroleum Gas or Biomass for Cooking and Effects on Blood Pressure".
Carotid intima-media thickness (CIMT)	**No**<br>In preparation
Brachial artery reactivity testing (BART)	**No**<br>In preparation

St. George Respiratory Questionnaire *and* SF-36 (Quality of Life)	**No**<br>In preparation
Household expenditures for fuel and healthcare	**No**<br>In preparation
Household time/activity	**No**<br>In preparation
Chronic disease biomarkers in the older adult woman	**No**<br>Awaiting final biomarker laboratory analyses

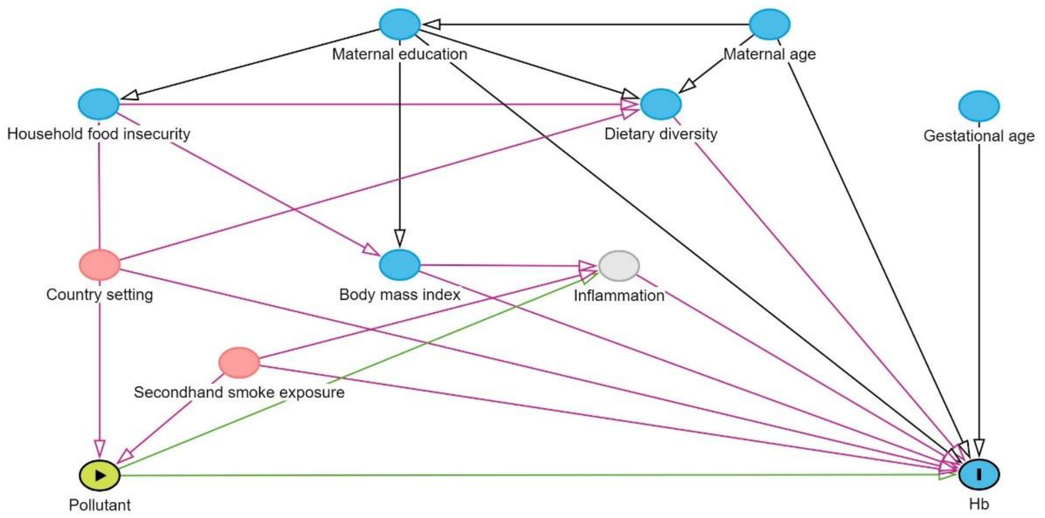

Figure S1. Directed acyclic graph of hypothesized relationships between household air pollution and hemoglobin (Hb) concentration.

This figure was created using Daggity (Johannes Textor, Benito van der Zander, Mark K. Gilthorpe, Maciej Liskiewicz, George T.H. Ellison. Robust causal inference using directed acyclic graphs: the R package 'dagitty'. *International Journal of Epidemiology* 45(6):1887-1894, 2016.)

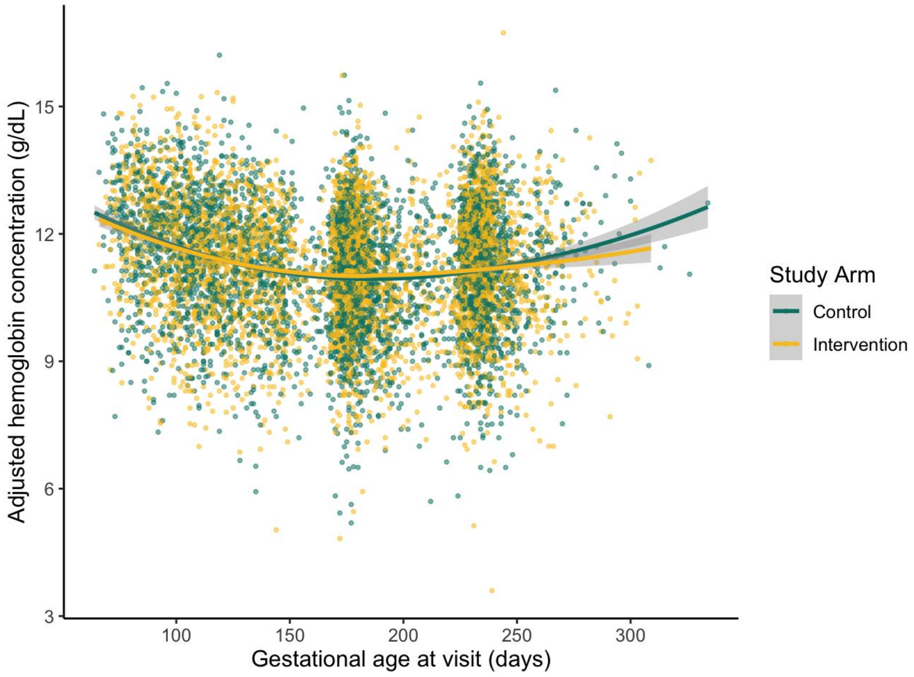

Figure S2. Adjusted hemoglobin level by gestational age at visit (in days) between the control and intervention groups. Source data are provided as a Source Data file.

Table S2. Summary of 24-hour personal $PM_{2.5}$, BC, and CO exposures by study arm and visit

	Control		Intervention	
	**N**	**Median [IQR]**	**N**	**Median [IQR]**
$PM_{2.5}$ ($\mu g/m^3$)				
Baseline	1411	83.2 [45.9, 142]	1396	81.7 [45.9, 151]
Visit 1	1238	72.1 [38.6, 126]	1282	24.1 [15.0, 39.5]
Visit 2	1127	69.7 [36.6, 131]	1170	23.7 [15.0, 39.6]
BC ($\mu g/m^3$)				
Baseline	1264	10.8 [6.84, 15.5]	1263	10.6 [6.23, 15.3]
Visit 1	1178	9.74 [5.31, 14.4]	1223	2.68 [1.62, 4.71]
Visit 2	1070	9.57 [5.22, 13.7]	1128	2.83 [1.7, 4.84]
CO (ppm)				
Baseline	1439	1.18 [0.50, 2.53]	1426	1.32 [0.48, 2.99]
Visit 1	1299	1.06 [0.40, 2.52]	1311	0.17 [0.03, 0.70]
Visit 2	1203	1.06 [0.34, 2.28]	1221	0.19 [0.03, 0.75]

Note: Summary based on all valid exposure measurements from the 3178 participants included in the analysis.

Table S3. Exposure-response analysis results at baseline – all IRCs

Exposures	Model Type	Estimate	p-value	95% CI	AIC
Crude Associations					
PM2.5	Linear	0.0004	0.1074	(-0.0001, 0.0008)	9895
	Log linear	0.1059	0.0007	(0.0447, 0.167)	9886
	Categorical [Ref. Quartile 1]				
	Quartile 2	-0.0863	0.2665	(-0.2385, 0.0659)	9900
	Quartile 3	-0.0967	0.2130	(-0.2488, 0.0555)	9900
	Quartile 4	-0.0465	0.5483	(-0.1984, 0.1054)	9900
BC	Linear	0.0005	0.8525	(-0.005, 0.006)	8888
	Log linear	0.1336	0.0003	(0.0613, 0.206)	8875
	Categorical [Ref. Quartile 1]				
	Quartile 2	-0.0157	0.8475	(-0.1754, 0.1441)	8891
	Quartile 3	-0.0396	0.6277	(-0.1994, 0.1203)	8891
	Quartile 4	0.0190	0.8149	(-0.1402, 0.1783)	8891
CO	Linear	0.0205	0.0016	(0.0078, 0.0332)	10140
	Log linear	0.0739	0.0000	(0.0404, 0.1073)	10131
	Categorical [Ref. Quartile 1]				
	Quartile 2	0.0434	0.5740	(-0.108, 0.1949)	10151
	Quartile 3	-0.0317	0.6823	(-0.1833, 0.12)	10151
	Quartile 4	0.1018	0.1883	(-0.0498, 0.2534)	10151
Adjusted Associations					
PM2.5	Linear	0.0000	0.9789	(-0.0004, 0.0004)	8919
	Log linear	0.0016	0.9550	(-0.0539, 0.0571)	8919
	Categorical [Ref. Quartile 1]				
	Quartile 2	-0.0692	0.2962	(-0.199, 0.0606)	8921
	Quartile 3	-0.0560	0.3997	(-0.1863, 0.0743)	8921
	Quartile 4	-0.0026	0.9687	(-0.1333, 0.128)	8921
BC	Linear	0.0009	0.7004	(-0.0038, 0.0056)	7968
	Log linear	0.0409	0.2085	(-0.0228, 0.1045)	7967
	Categorical [Ref. Quartile 1]				
	Quartile 2	0.0170	0.8049	(-0.1179, 0.1519)	7971
	Quartile 3	-0.0167	0.8097	(-0.1523, 0.119)	7971
	Quartile 4	0.0499	0.4725	(-0.0862, 0.1859)	7971
CO	Linear	0.0184	0.0011	(0.0074, 0.0293)	9159
	Log linear	0.0338	0.0231	(0.0047, 0.063)	9165
	Categorical [Ref. Quartile 1]				
	Quartile 2	0.0533	0.4177	(-0.0756, 0.1823)	9170
	Quartile 3	0.0025	0.9699	(-0.1269, 0.1318)	9170
	Quartile 4	0.1116	0.0915	(-0.018, 0.2411)	9170

Note: All adjusted exposure-response models controlled for maternal age, mother's highest education level, BMI at baseline, household food insecurity, mother's diet diversity, exposure to secondhand smoke, gestational age at the hemoglobin measurement and IRC. Estimates were obtained from multiple linear regression. Two-sided t-tests were used. No adjustment was made for multiple comparisons.

Table S4. Exposure-response analysis results at baseline – Guatemala

Exposures	Model Type	Estimate	p-value	95% CI	AIC
Crude Associations					
PM2.5	Linear	-0.0002	0.5040	(-0.0008, 0.0004)	2101
	Log linear	-0.0546	0.2616	(-0.1499, 0.0407)	2100
	Categorical [Ref. Quartile 1]				
	Quartile 2	-0.1046	0.3265	(-0.3134, 0.1042)	2103
	Quartile 3	-0.1255	0.2384	(-0.3341, 0.083)	2103
	Quartile 4	-0.1378	0.1974	(-0.3472, 0.0715)	2103
BC	Linear	-0.0093	0.0279	(-0.0176, -0.001)	1927
	Log linear	-0.0988	0.2275	(-0.259, 0.0615)	1931
	Categorical [Ref. Quartile 1]				
	Quartile 2	-0.0923	0.4054	(-0.3097, 0.125)	1935
	Quartile 3	-0.0067	0.9520	(-0.2244, 0.211)	1935
	Quartile 4	-0.0975	0.3788	(-0.3146, 0.1195)	1935
CO	Linear	-0.0033	0.7865	(-0.027, 0.0204)	2173
	Log linear	-0.0127	0.6297	(-0.0644, 0.039)	2173
	Categorical [Ref. Quartile 1]				
	Quartile 2	-0.0116	0.9122	(-0.2175, 0.1943)	2176
	Quartile 3	-0.0623	0.5535	(-0.2682, 0.1436)	2176
	Quartile 4	-0.1309	0.2125	(-0.3366, 0.0747)	2176
Adjusted Associations					
PM2.5	Linear	-0.0001	0.8569	(-0.0006, 0.0005)	2011
	Log linear	-0.0284	0.5455	(-0.1206, 0.0637)	2011
	Categorical [Ref. Quartile 1]				
	Quartile 2	-0.0436	0.6688	(-0.2431, 0.156)	2014
	Quartile 3	-0.0765	0.4551	(-0.2771, 0.1241)	2014
	Quartile 4	-0.0884	0.3921	(-0.2908, 0.114)	2014
BC	Linear	-0.0072	0.0759	(-0.0151, 0.0007)	1848
	Log linear	-0.0584	0.4552	(-0.2115, 0.0947)	1851
	Categorical [Ref. Quartile 1]				
	Quartile 2	-0.0118	0.9116	(-0.2207, 0.197)	1854
	Quartile 3	0.0292	0.7844	(-0.1798, 0.2382)	1854
	Quartile 4	-0.0678	0.5224	(-0.2753, 0.1398)	1854
CO	Linear	-0.0026	0.8220	(-0.0255, 0.0203)	2089
	Log linear	-0.0037	0.8842	(-0.0541, 0.0466)	2089
	Categorical [Ref. Quartile 1]				
	Quartile 2	-0.0164	0.8705	(-0.2137, 0.1809)	2092
	Quartile 3	-0.0579	0.5699	(-0.2574, 0.1417)	2092
	Quartile 4	-0.0818	0.4236	(-0.2821, 0.1185)	2092

Note: All adjusted exposure-response models controlled for maternal age, mother's highest education level, BMI at baseline, household food insecurity, mother's diet diversity, exposure to secondhand smoke, gestational age at the hemoglobin measurement. Estimates were obtained from multiple linear regression. Two-sided t-tests were used. No adjustment was made for multiple comparisons.

Table S5. Exposure-response analysis results at baseline – India

Exposures	Model Type	Estimate	p-value	95% CI	AIC
Crude Associations					
PM2.5	Linear	-0.0001	0.6674	(-0.0007, 0.0005)	2292
	Log linear	0.0388	0.4921	(-0.0719, 0.1496)	2291
	Categorical [Ref. Quartile 1]				
	Quartile 2	0.0777	0.5511	(-0.1776, 0.3329)	2294
	Quartile 3	0.0591	0.6506	(-0.1965, 0.3147)	2294
	Quartile 4	0.1528	0.2410	(-0.1024, 0.4081)	2294
BC	Linear	0.0052	0.2160	(-0.003, 0.0133)	2241
	Log linear	0.0971	0.0835	(-0.0127, 0.2069)	2239
	Categorical [Ref. Quartile 1]				
	Quartile 2	0.2497	0.0562	(-0.0062, 0.5056)	2239
	Quartile 3	0.0162	0.9014	(-0.2397, 0.2721)	2239
	Quartile 4	0.2792	0.0328	(0.0233, 0.5351)	2239
CO	Linear	0.0201	0.1569	(-0.0077, 0.0478)	2385
	Log linear	0.0467	0.0582	(-0.0016, 0.0949)	2383
	Categorical [Ref. Quartile 1]				
	Quartile 2	0.1304	0.3059	(-0.1191, 0.38)	2389
	Quartile 3	0.1739	0.1723	(-0.0756, 0.4234)	2389
	Quartile 4	0.1424	0.2637	(-0.1071, 0.3919)	2389
Adjusted Associations					
PM2.5	Linear	0.0000	0.9151	(-0.0007, 0.0006)	2288
	Log linear	0.0632	0.2710	(-0.0492, 0.1756)	2286
	Categorical [Ref. Quartile 1]				
	Quartile 2	0.0649	0.6223	(-0.1931, 0.3228)	2289
	Quartile 3	0.0762	0.5621	(-0.1812, 0.3336)	2289
	Quartile 4	0.2012	0.1285	(-0.0579, 0.4604)	2289
BC	Linear	0.0061	0.1507	(-0.0022, 0.0144)	2239
	Log linear	0.1133	0.0522	(-0.0009, 0.2275)	2237
	Categorical [Ref. Quartile 1]				
	Quartile 2	0.2574	0.0496	(0.0009, 0.5138)	2236
	Quartile 3	0.0157	0.9061	(-0.2451, 0.2765)	2236
	Quartile 4	0.3140	0.0195	(0.0511, 0.577)	2236
CO	Linear	0.0176	0.2165	(-0.0103, 0.0454)	2383
	Log linear	0.0489	0.0492	(0.0002, 0.0975)	2381
	Categorical [Ref. Quartile 1]				
	Quartile 2	0.1293	0.3102	(-0.1203, 0.3789)	2386
	Quartile 3	0.1904	0.1356	(-0.0594, 0.4402)	2386
	Quartile 4	0.1480	0.2507	(-0.1043, 0.4004)	2386

Note: All adjusted exposure-response models controlled for maternal age, mother's highest education level, BMI at baseline, household food insecurity, mother's diet diversity, exposure to secondhand smoke, gestational age at the hemoglobin measurement. Estimates were obtained from multiple linear regression. Two-sided t-tests were used. No adjustment was made for multiple comparisons.

Table S6. Exposure-response analysis results at baseline – Peru

Exposures	Model Type	Estimate	p-value	95% CI	AIC
Crude Associations					
PM2.5	Linear	0.0005	0.2894	(-0.0004, 0.0013)	2044
	Log linear	0.0507	0.2846	(-0.0421, 0.1435)	2044
	Categorical [Ref. Quartile 1]				
	Quartile 2	0.0832	0.5374	(-0.181, 0.3473)	2042
	Quartile 3	-0.0904	0.5024	(-0.3546, 0.1737)	2042
	Quartile 4	0.2471	0.0667	(-0.0166, 0.5109)	2042
BC	Linear	0.0047	0.2851	(-0.0039, 0.0132)	1846
	Log linear	0.0750	0.1284	(-0.0215, 0.1714)	1845
	Categorical [Ref. Quartile 1]				
	Quartile 2	0.1832	0.1977	(-0.0952, 0.4617)	1849
	Quartile 3	0.1255	0.3782	(-0.1534, 0.4044)	1849
	Quartile 4	0.1898	0.1790	(-0.0867, 0.4664)	1849
CO	Linear	0.0227	0.0081	(0.0059, 0.0394)	2111
	Log linear	0.0351	0.2672	(-0.0268, 0.097)	2117
	Categorical [Ref. Quartile 1]				
	Quartile 2	0.1614	0.2536	(-0.1155, 0.4383)	2117
	Quartile 3	-0.0783	0.5795	(-0.3552, 0.1986)	2117
	Quartile 4	0.1880	0.1871	(-0.091, 0.4671)	2117
Adjusted Associations					
PM2.5	Linear	0.0005	0.2089	(-0.0003, 0.0013)	1938
	Log linear	0.0490	0.2736	(-0.0387, 0.1367)	1938
	Categorical [Ref. Quartile 1]				
	Quartile 2	0.0443	0.7262	(-0.2036, 0.2923)	1938
	Quartile 3	-0.1005	0.4299	(-0.35, 0.1489)	1938
	Quartile 4	0.1975	0.1225	(-0.0528, 0.4477)	1938
BC	Linear	0.0042	0.3102	(-0.0039, 0.0122)	1743
	Log linear	0.0717	0.1240	(-0.0195, 0.1628)	1742
	Categorical [Ref. Quartile 1]				
	Quartile 2	0.1669	0.2113	(-0.0945, 0.4283)	1746
	Quartile 3	0.1209	0.3674	(-0.1418, 0.3835)	1746
	Quartile 4	0.1638	0.2206	(-0.098, 0.4256)	1746
CO	Linear	0.0241	0.0031	(0.0082, 0.0401)	2019
	Log linear	0.0522	0.0870	(-0.0075, 0.1118)	2025
	Categorical [Ref. Quartile 1]				
	Quartile 2	0.1408	0.3000	(-0.1252, 0.4068)	2026
	Quartile 3	-0.0174	0.8982	(-0.2836, 0.2488)	2026
	Quartile 4	0.2526	0.0668	(-0.017, 0.5223)	2026

Note: All adjusted exposure-response models controlled for maternal age, mother's highest education level, BMI at baseline, household food insecurity, mother's diet diversity, exposure to secondhand smoke, gestational age at the hemoglobin measurement. Estimates were obtained from multiple linear regression. Two-sided t-tests were used. No adjustment was made for multiple comparisons.

Table S7. Exposure-response analysis results at baseline – Rwanda

Exposures	Model Type	Estimate	p-value	95% CI	AIC
Crude Associations					
PM2.5	Linear	-0.0005	0.3793	(-0.0017, 0.0006)	2576
	Log linear	-0.1783	0.0239	(-0.3327, -0.0239)	2571
	Categorical [Ref. Quartile 1]				
	Quartile 2	-0.3748	0.0220	(-0.695, -0.0547)	2572
	Quartile 3	-0.2285	0.1617	(-0.5481, 0.0912)	2572
	Quartile 4	-0.4134	0.0109	(-0.7308, -0.096)	2572
BC	Linear	-0.0079	0.2919	(-0.0226, 0.0068)	2044
	Log linear	-0.2139	0.0424	(-0.42, -0.0078)	2041
	Categorical [Ref. Quartile 1]				
	Quartile 2	-0.4672	0.0108	(-0.8252, -0.1092)	2042
	Quartile 3	-0.3256	0.0747	(-0.683, 0.0318)	2042
	Quartile 4	-0.3588	0.0488	(-0.715, -0.0027)	2042
CO	Linear	0.0263	0.0573	(-0.0008, 0.0534)	2587
	Log linear	0.0401	0.3320	(-0.0409, 0.1212)	2590
	Categorical [Ref. Quartile 1]				
	Quartile 2	-0.0878	0.5913	(-0.408, 0.2325)	2588
	Quartile 3	-0.1547	0.3461	(-0.4764, 0.1669)	2588
	Quartile 4	0.2218	0.1739	(-0.0975, 0.5412)	2588
Adjusted Associations					
PM2.5	Linear	-0.0001	0.8373	(-0.0013, 0.0011)	2548
	Log linear	-0.0823	0.3257	(-0.2463, 0.0817)	2547
	Categorical [Ref. Quartile 1]				
	Quartile 2	-0.3822	0.0221	(-0.7087, -0.0556)	2546
	Quartile 3	-0.0980	0.5581	(-0.4258, 0.2298)	2546
	Quartile 4	-0.2496	0.1396	(-0.5805, 0.0812)	2546
BC	Linear	0.0000	0.9969	(-0.0152, 0.0151)	2022
	Log linear	-0.0881	0.4274	(-0.3054, 0.1293)	2021
	Categorical [Ref. Quartile 1]				
	Quartile 2	-0.3461	0.0619	(-0.7086, 0.0164)	2022
	Quartile 3	-0.1803	0.3414	(-0.5514, 0.1908)	2022
	Quartile 4	-0.1508	0.4258	(-0.5215, 0.22)	2022
CO	Linear	0.0229	0.0952	(-0.0039, 0.0496)	2549
	Log linear	0.0375	0.3605	(-0.0428, 0.1178)	2551
	Categorical [Ref. Quartile 1]				
	Quartile 2	-0.0098	0.9519	(-0.3272, 0.3076)	2553
	Quartile 3	-0.0972	0.5485	(-0.4144, 0.2201)	2553
	Quartile 4	0.1893	0.2447	(-0.1293, 0.5079)	2553

Note: All adjusted exposure-response models controlled for maternal age, mother's highest education level, BMI at baseline, household food insecurity, mother's diet diversity, exposure to secondhand smoke, gestational age at the hemoglobin measurement. Estimates were obtained from multiple linear regression. Two-sided t-tests were used. No adjustment was made for multiple comparisons.

Table S8. Exposure-response analysis results at post-randomization follow up visit 1 – all IRCs

Exposures	Model Type	Estimate	p-value	95% CI	AIC
Crude Associations					
PM2.5	Linear	0.0004	0.2351	(-0.0002, 0.001)	8697
	Log linear	0.0447	0.1410	(-0.0148, 0.1041)	8696
	Categorical [Ref. Quartile 1]				
	Quartile 2	-0.0450	0.5743	(-0.2019, 0.1119)	8698
	Quartile 3	-0.0611	0.4447	(-0.2177, 0.0955)	8698
	Quartile 4	-0.1564	0.0497	(-0.3126, -0.0003)	8698
BC	Linear	0.0047	0.1736	(-0.0021, 0.0114)	8290
	Log linear	0.1395	0.0000	(0.0786, 0.2004)	8272
	Categorical [Ref. Quartile 1]				
	Quartile 2	0.0957	0.2420	(-0.0646, 0.2559)	8291
	Quartile 3	-0.0295	0.7189	(-0.1899, 0.131)	8291
	Quartile 4	-0.0885	0.2796	(-0.249, 0.0719)	8291
CO	Linear	0.0043	0.6267	(-0.013, 0.0216)	9009
	Log linear	0.0309	0.0110	(0.0071, 0.0548)	9003
	Categorical [Ref. Quartile 1]				
	Quartile 2	-0.0115	0.8836	(-0.1651, 0.1421)	9011
	Quartile 3	-0.1089	0.1658	(-0.263, 0.0451)	9011
	Quartile 4	0.0075	0.9239	(-0.1457, 0.1607)	9011
Adjusted Associations					
PM2.5	Linear	-0.0001	0.7190	(-0.0007, 0.0005)	8111
	Log linear	-0.0345	0.2348	(-0.0915, 0.0224)	8110
	Categorical [Ref. Quartile 1]				
	Quartile 2	-0.0323	0.6536	(-0.1735, 0.1089)	8113
	Quartile 3	-0.0496	0.4920	(-0.191, 0.0918)	8113
	Quartile 4	-0.1036	0.1544	(-0.2461, 0.0389)	8113
BC	Linear	-0.0018	0.5727	(-0.008, 0.0044)	7723
	Log linear	-0.0386	0.2041	(-0.0983, 0.021)	7721
	Categorical [Ref. Quartile 1]				
	Quartile 2	0.0979	0.1825	(-0.046, 0.2417)	7723
	Quartile 3	-0.0101	0.8915	(-0.1546, 0.1345)	7723
	Quartile 4	-0.0407	0.5839	(-0.1861, 0.1048)	7723
CO	Linear	0.0158	0.0477	(0.0002, 0.0315)	8374
	Log linear	0.0022	0.8451	(-0.0198, 0.0242)	8378
	Categorical [Ref. Quartile 1]				
	Quartile 2	-0.0006	0.9933	(-0.1378, 0.1367)	8379
	Quartile 3	-0.0950	0.1761	(-0.2325, 0.0426)	8379
	Quartile 4	0.0251	0.7193	(-0.1119, 0.1622)	8379

Note: All adjusted exposure-response models controlled for maternal age, mother's highest education level, BMI at baseline, household food insecurity, mother's diet diversity, exposure to secondhand smoke, gestational age at the hemoglobin measurement and IRC. Estimates were obtained from multiple linear regression. Two-sided t-tests were used. No adjustment was made for multiple comparisons.

Table S9. Exposure-response analysis results at post-randomization follow up visit 1 – Guatemala

Exposures	Model Type	Estimate	p-value	95% CI	AIC
Crude Associations					
PM2.5	Linear	0.0004	0.3481	(-0.0004, 0.0012)	2091
	Log linear	0.0197	0.6221	(-0.0588, 0.0983)	2092
	Categorical [Ref. Quartile 1]				
	Quartile 2	0.0378	0.7426	(-0.1876, 0.2631)	2094
	Quartile 3	-0.0955	0.4063	(-0.3209, 0.1298)	2094
	Quartile 4	0.0417	0.7172	(-0.1837, 0.267)	2094
BC	Linear	0.0010	0.8448	(-0.0091, 0.0111)	2061
	Log linear	-0.0046	0.9343	(-0.113, 0.1038)	2061
	Categorical [Ref. Quartile 1]				
	Quartile 2	-0.0770	0.5068	(-0.3041, 0.1501)	2062
	Quartile 3	-0.1869	0.1096	(-0.4156, 0.0418)	2062
	Quartile 4	0.0074	0.9496	(-0.2213, 0.2361)	2062
CO	Linear	-0.0110	0.6010	(-0.052, 0.0301)	2089
	Log linear	-0.0158	0.4041	(-0.0528, 0.0213)	2089
	Categorical [Ref. Quartile 1]				
	Quartile 2	0.0638	0.5597	(-0.1506, 0.2783)	2092
	Quartile 3	-0.0342	0.7549	(-0.2489, 0.1805)	2092
	Quartile 4	-0.0561	0.6075	(-0.2703, 0.158)	2092
Adjusted Associations					
PM2.5	Linear	0.0004	0.3022	(-0.0004, 0.0013)	2080
	Log linear	0.0252	0.5379	(-0.0549, 0.1053)	2081
	Categorical [Ref. Quartile 1]				
	Quartile 2	0.0331	0.7757	(-0.1944, 0.2606)	2083
	Quartile 3	-0.0986	0.3972	(-0.3269, 0.1296)	2083
	Quartile 4	0.0573	0.6255	(-0.1727, 0.2874)	2083
BC	Linear	0.0017	0.7492	(-0.0085, 0.0119)	2052
	Log linear	0.0056	0.9203	(-0.1049, 0.1162)	2052
	Categorical [Ref. Quartile 1]				
	Quartile 2	-0.0547	0.6387	(-0.2829, 0.1735)	2052
	Quartile 3	-0.1728	0.1424	(-0.4035, 0.0578)	2052
	Quartile 4	0.0284	0.8106	(-0.2035, 0.2602)	2052
CO	Linear	-0.0103	0.6241	(-0.0514, 0.0309)	2079
	Log linear	-0.0157	0.4104	(-0.0532, 0.0217)	2079
	Categorical [Ref. Quartile 1]				
	Quartile 2	0.0511	0.6428	(-0.1648, 0.267)	2082
	Quartile 3	-0.0228	0.8360	(-0.2388, 0.1932)	2082
	Quartile 4	-0.0632	0.5669	(-0.2796, 0.1531)	2082

Note: All adjusted exposure-response models controlled for maternal age, mother's highest education level, BMI at baseline, household food insecurity, mother's diet diversity, exposure to secondhand smoke, gestational age at the hemoglobin measurement. Estimates were obtained from multiple linear regression. Two-sided t-tests were used. No adjustment was made for multiple comparisons.

Table S10. Exposure-response analysis results at post-randomization follow up visit 1 – India

Exposures	Model Type	Estimate	p-value	95% CI	AIC
Crude Associations					
PM2.5	Linear	-0.0006	0.2486	(-0.0017, 0.0004)	2030
	Log linear	-0.1206	0.0356	(-0.2328, -0.0084)	2027
	Categorical [Ref. Quartile 1]				
	Quartile 2	-0.1631	0.2545	(-0.4435, 0.1172)	2031
	Quartile 3	-0.2078	0.1474	(-0.4887, 0.073)	2031
	Quartile 4	-0.3084	0.0309	(-0.5878, -0.0289)	2031
BC	Linear	-0.0136	0.0321	(-0.026, -0.0012)	1971
	Log linear	-0.1002	0.0352	(-0.1933, -0.0071)	1971
	Categorical [Ref. Quartile 1]				
	Quartile 2	0.1424	0.3246	(-0.1407, 0.4255)	1972
	Quartile 3	-0.0634	0.6602	(-0.3461, 0.2192)	1972
	Quartile 4	-0.2523	0.0817	(-0.5359, 0.0312)	1972
CO	Linear	0.0120	0.5091	(-0.0235, 0.0474)	2245
	Log linear	-0.0304	0.0733	(-0.0635, 0.0028)	2242
	Categorical [Ref. Quartile 1]				
	Quartile 2	-0.0566	0.6680	(-0.3154, 0.2021)	2241
	Quartile 3	-0.3414	0.0104	(-0.6016, -0.0811)	2241
	Quartile 4	-0.2092	0.1134	(-0.468, 0.0495)	2241
Adjusted Associations					
PM2.5	Linear	-0.0003	0.5625	(-0.0014, 0.0008)	2027
	Log linear	-0.0999	0.0841	(-0.2131, 0.0133)	2024
	Categorical [Ref. Quartile 1]				
	Quartile 2	-0.1916	0.1801	(-0.4714, 0.0883)	2027
	Quartile 3	-0.2097	0.1411	(-0.4886, 0.0692)	2027
	Quartile 4	-0.2858	0.0466	(-0.5666, -0.0049)	2027
BC	Linear	-0.0107	0.0948	(-0.0232, 0.0018)	1968
	Log linear	-0.0940	0.0508	(-0.1881, 0.0001)	1967
	Categorical [Ref. Quartile 1]				
	Quartile 2	0.1237	0.3899	(-0.1581, 0.4055)	1967
	Quartile 3	-0.1299	0.3732	(-0.4157, 0.1558)	1967
	Quartile 4	-0.2387	0.1003	(-0.523, 0.0455)	1967
CO	Linear	0.0128	0.4795	(-0.0227, 0.0484)	2240
	Log linear	-0.0274	0.1043	(-0.0605, 0.0056)	2237
	Categorical [Ref. Quartile 1]				
	Quartile 2	-0.0351	0.7890	(-0.2922, 0.222)	2237
	Quartile 3	-0.3192	0.0162	(-0.5788, -0.0596)	2237
	Quartile 4	-0.1866	0.1558	(-0.444, 0.0708)	2237

Note: All adjusted exposure-response models controlled for maternal age, mother's highest education level, BMI at baseline, household food insecurity, mother's diet diversity, exposure to secondhand smoke, gestational age at the hemoglobin measurement. Estimates were obtained from multiple linear regression. Two-sided t-tests were used. No adjustment was made for multiple comparisons.

Table S11. Exposure-response analysis results at post-randomization follow up visit 1 – Peru

Exposures	Model Type	Estimate	p-value	95% CI	AIC
Crude Associations					
PM2.5	Linear	-0.0001	0.8284	(-0.0014, 0.0011)	1687
	Log linear	-0.0519	0.3882	(-0.1697, 0.0659)	1686
	Categorical [Ref. Quartile 1]				
	Quartile 2	0.0333	0.8162	(-0.2476, 0.3143)	1690
	Quartile 3	0.0322	0.8211	(-0.2467, 0.3111)	1690
	Quartile 4	-0.0832	0.5587	(-0.3621, 0.1956)	1690
BC	Linear	-0.0081	0.2294	(-0.0212, 0.0051)	1557
	Log linear	-0.0528	0.3533	(-0.1643, 0.0586)	1557
	Categorical [Ref. Quartile 1]				
	Quartile 2	0.1804	0.2256	(-0.111, 0.4719)	1560
	Quartile 3	0.1934	0.1949	(-0.0987, 0.4854)	1560
	Quartile 4	0.0493	0.7400	(-0.2416, 0.3402)	1560
CO	Linear	0.0082	0.4169	(-0.0116, 0.028)	1610
	Log linear	0.0117	0.6430	(-0.0376, 0.061)	1610
	Categorical [Ref. Quartile 1]				
	Quartile 2	-0.0989	0.5051	(-0.3895, 0.1917)	1614
	Quartile 3	-0.0687	0.6439	(-0.3599, 0.2225)	1614
	Quartile 4	-0.0086	0.9536	(-0.2981, 0.2809)	1614
Adjusted Associations					
PM2.5	Linear	0.0001	0.8716	(-0.0012, 0.0014)	1660
	Log linear	-0.0171	0.7782	(-0.1361, 0.1019)	1660
	Categorical [Ref. Quartile 1]				
	Quartile 2	0.0319	0.8252	(-0.2514, 0.3153)	1664
	Quartile 3	-0.0142	0.9217	(-0.2968, 0.2684)	1664
	Quartile 4	-0.0254	0.8604	(-0.3079, 0.2571)	1664
BC	Linear	-0.0051	0.4608	(-0.0185, 0.0084)	1540
	Log linear	-0.0197	0.7345	(-0.1333, 0.0939)	1540
	Categorical [Ref. Quartile 1]				
	Quartile 2	0.1337	0.3716	(-0.1593, 0.4267)	1543
	Quartile 3	0.1634	0.2756	(-0.13, 0.4569)	1543
	Quartile 4	0.1019	0.4969	(-0.1919, 0.3956)	1543
CO	Linear	0.0097	0.3415	(-0.0102, 0.0296)	1595
	Log linear	0.0151	0.5518	(-0.0346, 0.0649)	1595
	Categorical [Ref. Quartile 1]				
	Quartile 2	-0.1274	0.3937	(-0.42, 0.1651)	1598
	Quartile 3	-0.0712	0.6350	(-0.3652, 0.2228)	1598
	Quartile 4	0.0206	0.8903	(-0.2715, 0.3127)	1598

Note: All adjusted exposure-response models controlled for maternal age, mother's highest education level, BMI at baseline, household food insecurity, mother's diet diversity, exposure to secondhand smoke, gestational age at the hemoglobin measurement. Estimates were obtained from multiple linear regression. Two-sided t-tests were used. No adjustment was made for multiple comparisons.

Table S12. Exposure-response analysis results at post-randomization follow up visit 1 – Rwanda

Exposures	Model Type	Estimate	p-value	95% CI	AIC
Crude Associations					
PM2.5	Linear	-0.0009	0.1677	(-0.0023, 0.0004)	2292
	Log linear	-0.1377	0.0833	(-0.2932, 0.0179)	2291
	Categorical [Ref. Quartile 1]				
	Quartile 2	-0.1035	0.5521	(-0.4446, 0.2375)	2294
	Quartile 3	0.0276	0.8735	(-0.3123, 0.3676)	2294
	Quartile 4	-0.2974	0.0850	(-0.6353, 0.0405)	2294
BC	Linear	0.0018	0.7973	(-0.0118, 0.0154)	2121
	Log linear	-0.0687	0.4631	(-0.2519, 0.1146)	2120
	Categorical [Ref. Quartile 1]				
	Quartile 2	0.1539	0.3918	(-0.198, 0.5058)	2122
	Quartile 3	-0.0079	0.9648	(-0.3586, 0.3428)	2122
	Quartile 4	-0.1671	0.3507	(-0.5178, 0.1836)	2122
CO	Linear	0.0544	0.0263	(0.0065, 0.1023)	2393
	Log linear	0.0800	0.0192	(0.0132, 0.1468)	2392
	Categorical [Ref. Quartile 1]				
	Quartile 2	0.0244	0.8857	(-0.308, 0.3568)	2397
	Quartile 3	0.0112	0.9476	(-0.3218, 0.3441)	2397
	Quartile 4	0.3128	0.0639	(-0.0176, 0.6432)	2397
Adjusted Associations					
PM2.5	Linear	-0.0006	0.4283	(-0.0019, 0.0008)	2281
	Log linear	-0.0520	0.5319	(-0.215, 0.111)	2281
	Categorical [Ref. Quartile 1]				
	Quartile 2	-0.0175	0.9206	(-0.3613, 0.3263)	2283
	Quartile 3	0.1697	0.3395	(-0.1783, 0.5177)	2283
	Quartile 4	-0.1033	0.5666	(-0.4566, 0.2499)	2283
BC	Linear	0.0066	0.3507	(-0.0073, 0.0205)	2107
	Log linear	0.0331	0.7342	(-0.1577, 0.2239)	2108
	Categorical [Ref. Quartile 1]				
	Quartile 2	0.2415	0.1804	(-0.1114, 0.5945)	2110
	Quartile 3	0.0969	0.5941	(-0.2593, 0.4531)	2110
	Quartile 4	0.0316	0.8649	(-0.3323, 0.3955)	2110
CO	Linear	0.0537	0.0281	(0.0059, 0.1015)	2383
	Log linear	0.0891	0.0093	(0.0222, 0.1561)	2381
	Categorical [Ref. Quartile 1]				
	Quartile 2	0.1284	0.4505	(-0.2049, 0.4618)	2387
	Quartile 3	0.0725	0.6709	(-0.2618, 0.4068)	2387
	Quartile 4	0.3724	0.0284	(0.0401, 0.7048)	2387

Note: All adjusted exposure-response models controlled for maternal age, mother's highest education level, BMI at baseline, household food insecurity, mother's diet diversity, exposure to secondhand smoke, gestational age at the hemoglobin measurement. Estimates were obtained from multiple linear regression. Two-sided t-tests were used. No adjustment was made for multiple comparisons.

Table S13. Exposure-response analysis results at post-randomization follow up visit 2 – all IRCs

Exposures	Model Type	Estimate	p-value	95% CI	AIC
Crude Associations					
PM2.5	Linear	0.0006	0.0961	(-0.0001, 0.0012)	7955
	Log linear	0.1265	0.0001	(0.0638, 0.1891)	7942
	Categorical [Ref. Quartile 1]				
	Quartile 2	-0.0679	0.4258	(-0.2349, 0.0991)	7960
	Quartile 3	-0.0016	0.9849	(-0.1682, 0.165)	7960
	Quartile 4	-0.0784	0.3563	(-0.2449, 0.0881)	7960
BC	Linear	0.0088	0.0116	(0.002, 0.0156)	7629
	Log linear	0.2138	0.0000	(0.148, 0.2797)	7595
	Categorical [Ref. Quartile 1]				
	Quartile 2	-0.0139	0.8735	(-0.1852, 0.1574)	7637
	Quartile 3	-0.1170	0.1792	(-0.2875, 0.0536)	7637
	Quartile 4	-0.0453	0.6033	(-0.2159, 0.1254)	7637
CO	Linear	0.0088	0.3511	(-0.0097, 0.0274)	8410
	Log linear	0.0260	0.0417	(0.001, 0.051)	8407
	Categorical [Ref. Quartile 1]				
	Quartile 2	0.0340	0.6812	(-0.1282, 0.1963)	8410
	Quartile 3	-0.1331	0.1074	(-0.2951, 0.0289)	8410
	Quartile 4	-0.0143	0.8630	(-0.1768, 0.1482)	8410
Adjusted Associations					
PM2.5	Linear	0.0001	0.6400	(-0.0004, 0.0007)	7372
	Log linear	0.0130	0.6695	(-0.0467, 0.0727)	7372
	Categorical [Ref. Quartile 1]				
	Quartile 2	-0.0470	0.5347	(-0.1955, 0.1014)	7375
	Quartile 3	0.0504	0.5064	(-0.0983, 0.1991)	7375
	Quartile 4	-0.0267	0.7274	(-0.1765, 0.1232)	7375
BC	Linear	0.0037	0.2331	(-0.0024, 0.0099)	7073
	Log linear	0.0147	0.6540	(-0.0495, 0.0789)	7074
	Categorical [Ref. Quartile 1]				
	Quartile 2	-0.0104	0.8930	(-0.1624, 0.1415)	7077
	Quartile 3	-0.0797	0.3033	(-0.2313, 0.072)	7077
	Quartile 4	-0.0035	0.9640	(-0.1566, 0.1495)	7077
CO	Linear	0.0223	0.0088	(0.0057, 0.039)	7810
	Log linear	0.0034	0.7724	(-0.0196, 0.0264)	7817
	Categorical [Ref. Quartile 1]				
	Quartile 2	0.0494	0.5038	(-0.0954, 0.1941)	7816
	Quartile 3	-0.1115	0.1305	(-0.256, 0.033)	7816
	Quartile 4	0.0100	0.8931	(-0.1352, 0.1551)	7816

Note: All adjusted exposure-response models controlled for maternal age, mother's highest education level, BMI at baseline, household food insecurity, mother's diet diversity, exposure to secondhand smoke, gestational age at the hemoglobin measurement and IRC. Estimates were obtained from multiple linear regression. Two-sided t-tests were used. No adjustment was made for multiple comparisons.

Table S14. Exposure-response analysis results at post-randomization follow up visit 2 – Guatemala

Exposures	Model Type	Estimate	p-value	95% CI	AIC
Crude Associations					
PM2.5	Linear	0.0004	0.3689	(-0.0005, 0.0014)	1864
	Log linear	0.0100	0.8126	(-0.0727, 0.0928)	1865
	Categorical [Ref. Quartile 1]				
	Quartile 2	-0.2808	0.0153	(-0.5071, -0.0545)	1862
	Quartile 3	-0.0621	0.5920	(-0.2891, 0.1649)	1862
	Quartile 4	-0.1182	0.3064	(-0.3444, 0.1081)	1862
BC	Linear	0.0015	0.7996	(-0.0101, 0.0131)	1832
	Log linear	-0.0037	0.9484	(-0.117, 0.1095)	1832
	Categorical [Ref. Quartile 1]				
	Quartile 2	-0.2706	0.0205	(-0.4988, -0.0424)	1829
	Quartile 3	-0.0038	0.9739	(-0.2313, 0.2237)	1829
	Quartile 4	-0.0540	0.6423	(-0.2819, 0.1738)	1829
CO	Linear	-0.0168	0.4573	(-0.0612, 0.0275)	1925
	Log linear	-0.0229	0.2208	(-0.0595, 0.0137)	1924
	Categorical [Ref. Quartile 1]				
	Quartile 2	-0.1072	0.3348	(-0.3248, 0.1105)	1926
	Quartile 3	-0.1546	0.1650	(-0.3725, 0.0634)	1926
	Quartile 4	-0.2010	0.0712	(-0.4189, 0.017)	1926
Adjusted Associations					
PM2.5	Linear	0.0006	0.2301	(-0.0004, 0.0015)	1844
	Log linear	0.0107	0.8002	(-0.0722, 0.0936)	1845
	Categorical [Ref. Quartile 1]				
	Quartile 2	-0.3384	0.0034	(-0.5639, -0.1129)	1839
	Quartile 3	-0.0677	0.5572	(-0.2935, 0.1581)	1839
	Quartile 4	-0.1316	0.2534	(-0.3571, 0.094)	1839
BC	Linear	0.0009	0.8786	(-0.0106, 0.0124)	1812
	Log linear	-0.0112	0.8464	(-0.1242, 0.1018)	1812
	Categorical [Ref. Quartile 1]				
	Quartile 2	-0.2750	0.0178	(-0.5019, -0.0481)	1809
	Quartile 3	-0.0058	0.9599	(-0.2321, 0.2205)	1809
	Quartile 4	-0.0674	0.5614	(-0.2947, 0.1599)	1809
CO	Linear	-0.0110	0.6248	(-0.0549, 0.033)	1902
	Log linear	-0.0203	0.2736	(-0.0567, 0.016)	1901
	Categorical [Ref. Quartile 1]				
	Quartile 2	-0.1008	0.3609	(-0.3169, 0.1153)	1904
	Quartile 3	-0.1451	0.1912	(-0.3624, 0.0723)	1904
	Quartile 4	-0.1782	0.1080	(-0.3951, 0.0388)	1904

Note: All adjusted exposure-response models controlled for maternal age, mother's highest education level, BMI at baseline, household food insecurity, mother's diet diversity, exposure to secondhand smoke, gestational age at the hemoglobin measurement. Estimates were obtained from multiple linear regression. Two-sided t-tests were used. No adjustment was made for multiple comparisons.

Table S15. Exposure-response analysis results at post-randomization follow up visit 2 – India

Exposures	Model Type	Estimate	p-value	95% CI	AIC
Crude Associations					
PM2.5	Linear	-0.0004	0.5530	(-0.0016, 0.0008)	1964
	Log linear	-0.0046	0.9420	(-0.1278, 0.1186)	1964
	Categorical [Ref. Quartile 1]				
	Quartile 2	0.1250	0.4493	(-0.1986, 0.4486)	1967
	Quartile 3	0.1157	0.4836	(-0.2078, 0.4393)	1967
	Quartile 4	0.0760	0.6438	(-0.2459, 0.3978)	1967
BC	Linear	-0.0020	0.7138	(-0.0125, 0.0086)	1928
	Log linear	-0.0295	0.5949	(-0.1381, 0.0791)	1927
	Categorical [Ref. Quartile 1]				
	Quartile 2	0.2777	0.0953	(-0.048, 0.6033)	1926
	Quartile 3	-0.1041	0.5305	(-0.4291, 0.221)	1926
	Quartile 4	0.0541	0.7428	(-0.2691, 0.3774)	1926
CO	Linear	-0.0125	0.5132	(-0.05, 0.025)	2143
	Log linear	-0.0169	0.3988	(-0.0561, 0.0223)	2143
	Categorical [Ref. Quartile 1]				
	Quartile 2	0.3249	0.0361	(0.0216, 0.6281)	2138
	Quartile 3	-0.1437	0.3525	(-0.4464, 0.159)	2138
	Quartile 4	-0.0349	0.8219	(-0.3386, 0.2688)	2138
Adjusted Associations					
PM2.5	Linear	-0.0001	0.8091	(-0.0014, 0.0011)	1952
	Log linear	0.0364	0.5625	(-0.0868, 0.1596)	1952
	Categorical [Ref. Quartile 1]				
	Quartile 2	0.1189	0.4659	(-0.2004, 0.4382)	1955
	Quartile 3	0.1609	0.3268	(-0.1605, 0.4823)	1955
	Quartile 4	0.1600	0.3255	(-0.1586, 0.4786)	1955
BC	Linear	0.0003	0.9561	(-0.0102, 0.0108)	1916
	Log linear	0.0105	0.8487	(-0.0977, 0.1188)	1916
	Categorical [Ref. Quartile 1]				
	Quartile 2	0.2475	0.1337	(-0.0755, 0.5705)	1915
	Quartile 3	-0.0422	0.7979	(-0.3647, 0.2803)	1915
	Quartile 4	0.1753	0.2866	(-0.1469, 0.4975)	1915
CO	Linear	-0.0060	0.7527	(-0.0433, 0.0313)	2128
	Log linear	-0.0122	0.5357	(-0.0509, 0.0264)	2128
	Categorical [Ref. Quartile 1]				
	Quartile 2	0.2998	0.0491	(0.0018, 0.5978)	2123
	Quartile 3	-0.1396	0.3616	(-0.4394, 0.1601)	2123
	Quartile 4	-0.0018	0.9905	(-0.303, 0.2993)	2123

Note: All adjusted exposure-response models controlled for maternal age, mother's highest education level, BMI at baseline, household food insecurity, mother's diet diversity, exposure to secondhand smoke, gestational age at the hemoglobin measurement. Estimates were obtained from multiple linear regression. Two-sided t-tests were used. No adjustment was made for multiple comparisons.

Table S16. Exposure-response analysis results at post-randomization follow up visit 2 – Peru

Exposures	Model Type	Estimate	p-value	95% CI	AIC
Crude Associations					
PM2.5	Linear	0.0007	0.1784	(-0.0003, 0.0018)	1519
	Log linear	0.0861	0.1914	(-0.0429, 0.2151)	1519
	Categorical [Ref. Quartile 1]				
	Quartile 2	0.1485	0.3667	(-0.1736, 0.4706)	1524
	Quartile 3	0.1358	0.4041	(-0.1829, 0.4545)	1524
	Quartile 4	0.1169	0.4772	(-0.2052, 0.439)	1524
BC	Linear	0.0176	0.0038	(0.0058, 0.0294)	1391
	Log linear	0.1292	0.0591	(-0.0046, 0.2629)	1395
	Categorical [Ref. Quartile 1]				
	Quartile 2	-0.1559	0.3673	(-0.4944, 0.1826)	1400
	Quartile 3	-0.2140	0.2105	(-0.5484, 0.1205)	1400
	Quartile 4	0.0567	0.7426	(-0.2817, 0.3952)	1400
CO	Linear	0.0368	0.0033	(0.0124, 0.0612)	1456
	Log linear	0.0529	0.0584	(-0.0017, 0.1076)	1461
	Categorical [Ref. Quartile 1]				
	Quartile 2	0.0542	0.7459	(-0.2734, 0.3818)	1467
	Quartile 3	-0.0737	0.6600	(-0.4021, 0.2546)	1467
	Quartile 4	0.1648	0.3270	(-0.1643, 0.4939)	1467
Adjusted Associations					
PM2.5	Linear	0.0007	0.2255	(-0.0004, 0.0017)	1485
	Log linear	0.0794	0.2259	(-0.049, 0.2078)	1485
	Categorical [Ref. Quartile 1]				
	Quartile 2	0.1674	0.3015	(-0.1498, 0.4845)	1488
	Quartile 3	0.2575	0.1067	(-0.0547, 0.5698)	1488
	Quartile 4	0.1183	0.4665	(-0.1999, 0.4365)	1488
BC	Linear	0.0171	0.0050	(0.0052, 0.0289)	1363
	Log linear	0.1257	0.0643	(-0.0071, 0.2585)	1368
	Categorical [Ref. Quartile 1]				
	Quartile 2	-0.0668	0.6942	(-0.3996, 0.266)	1374
	Quartile 3	-0.0704	0.6782	(-0.4025, 0.2618)	1374
	Quartile 4	0.1099	0.5200	(-0.2245, 0.4442)	1374
CO	Linear	0.0337	0.0054	(0.01, 0.0573)	1415
	Log linear	0.0496	0.0696	(-0.0038, 0.1031)	1420
	Categorical [Ref. Quartile 1]				
	Quartile 2	0.0552	0.7355	(-0.265, 0.3754)	1425
	Quartile 3	-0.0502	0.7592	(-0.371, 0.2706)	1425
	Quartile 4	0.1723	0.2918	(-0.1476, 0.4922)	1425

Note: All adjusted exposure-response models controlled for maternal age, mother's highest education level, BMI at baseline, household food insecurity, mother's diet diversity, exposure to secondhand smoke, gestational age at the hemoglobin measurement. Estimates were obtained from multiple linear regression. Two-sided t-tests were used. No adjustment was made for multiple comparisons.

Table S17. Exposure-response analysis results at post-randomization follow up visit 2 – Rwanda

Exposures	Model Type	Estimate	p-value	95% CI	AIC
Crude Associations					
PM2.5	Linear	-0.0012	0.1161	(-0.0028, 0.0003)	2039
	Log linear	-0.1381	0.0747	(-0.2897, 0.0135)	2038
	Categorical [Ref. Quartile 1]				
	Quartile 2	-0.1961	0.2376	(-0.5212, 0.129)	2041
	Quartile 3	-0.1421	0.3897	(-0.4655, 0.1814)	2041
	Quartile 4	-0.3403	0.0393	(-0.6632, -0.0174)	2041
BC	Linear	-0.0085	0.3390	(-0.0259, 0.0089)	1929
	Log linear	-0.1159	0.1994	(-0.2926, 0.0609)	1929
	Categorical [Ref. Quartile 1]				
	Quartile 2	0.0768	0.6568	(-0.2616, 0.4152)	1930
	Quartile 3	-0.1886	0.2742	(-0.5264, 0.1491)	1930
	Quartile 4	-0.2215	0.1975	(-0.5581, 0.115)	1930
CO	Linear	0.0347	0.0827	(-0.0044, 0.0739)	2285
	Log linear	0.0130	0.6764	(-0.0479, 0.0738)	2288
	Categorical [Ref. Quartile 1]				
	Quartile 2	-0.1202	0.4591	(-0.4382, 0.1978)	2289
	Quartile 3	-0.1539	0.3395	(-0.4695, 0.1617)	2289
	Quartile 4	0.0626	0.6995	(-0.255, 0.3801)	2289
Adjusted Associations					
PM2.5	Linear	-0.0006	0.4984	(-0.0022, 0.0011)	2030
	Log linear	-0.0719	0.3888	(-0.2353, 0.0915)	2030
	Categorical [Ref. Quartile 1]				
	Quartile 2	-0.1356	0.4221	(-0.4664, 0.1952)	2033
	Quartile 3	-0.0837	0.6238	(-0.4179, 0.2505)	2033
	Quartile 4	-0.2247	0.2066	(-0.5729, 0.1236)	2033
BC	Linear	-0.0041	0.6605	(-0.0221, 0.014)	1920
	Log linear	-0.0818	0.3927	(-0.2693, 0.1056)	1919
	Categorical [Ref. Quartile 1]				
	Quartile 2	0.0698	0.6895	(-0.2724, 0.412)	1921
	Quartile 3	-0.1859	0.2899	(-0.5299, 0.158)	1921
	Quartile 4	-0.1659	0.3620	(-0.5224, 0.1905)	1921
CO	Linear	0.0391	0.0512	(-0.0001, 0.0783)	2273
	Log linear	0.0204	0.5177	(-0.0413, 0.0821)	2276
	Categorical [Ref. Quartile 1]				
	Quartile 2	-0.0963	0.5565	(-0.4171, 0.2245)	2278
	Quartile 3	-0.1172	0.4694	(-0.4344, 0.2001)	2278
	Quartile 4	0.0936	0.5676	(-0.2272, 0.4144)	2278

Note: All adjusted exposure-response models controlled for maternal age, mother's highest education level, BMI at baseline, household food insecurity, mother's diet diversity, exposure to secondhand smoke, gestational age at the hemoglobin measurement. Estimates were obtained from multiple linear regression. Two-sided t-tests were used. No adjustment was made for multiple comparisons.

Table S18. Exposure-response analysis results from mixed-effects models – all IRCs

Exposures	Model Type	Estimate	p-value	95% CI	AIC
Crude Associations					
$PM_{2.5}$	Linear	0.0006	<0.0001*	(0.0003, 0.0009)	25020
	Log linear	0.1300	<0.0001*	(0.0998, 0.1602)	24959
	Categorical [Ref. Quartile 1]				
	Quartile 2	0.0546	0.1589	(-0.0214, 0.1307)	25014
	Quartile 3	0.1483	0.0002	(0.0707, 0.2259)	25014
	Quartile 4	0.1920	<0.0001*	(0.1120, 0.2719)	25014
BC	Linear	0.0086	<0.0001*	(0.0055, 0.0117)	23437
	Log linear	0.1712	<0.0001*	(0.1387, 0.2038)	23356
	Categorical [Ref. Quartile 1]				
	Quartile 2	0.0532	0.1885	(-0.0261, 0.1325)	23435
	Quartile 3	0.1967	<0.0001*	(0.1160, 0.2775)	23435
	Quartile 4	0.2175	<0.0001*	(0.1349, 0.3002)	23435
CO	Linear	0.0212	<0.0001*	(0.0135, 0.029)	25846
	Log linear	0.0443	<0.0001*	(0.0315, 0.0572)	25828
	Categorical [Ref. Quartile 1]				
	Quartile 2	0.0539	0.1515	(-0.0198, 0.1276)	25855
	Quartile 3	0.0687	0.0732	(-0.0064, 0.1438)	25855
	Quartile 4	0.2024	<0.0001*	(0.1257, 0.2792)	25855
Adjusted Associations					
$PM_{2.5}$	Linear	-0.0001	0.5840	(-0.0003, 0.0002)	23456
	Log linear	-0.0009	0.9525	(-0.0318, 0.0299)	23447
	Categorical [Ref. Quartile 1]				
	Quartile 2	-0.0339	0.3543	(-0.1057, 0.0378)	23458
	Quartile 3	-0.0325	0.3982	(-0.108, 0.043)	23458
	Quartile 4	-0.0365	0.3686	(-0.116, 0.0431)	23458
BC	Linear	0.0013	0.3965	(-0.0017, 0.0043)	21968
	Log linear	0.0152	0.3728	(-0.0182, 0.0487)	21963
	Categorical [Ref. Quartile 1]				
	Quartile 2	-0.0397	0.2988	(-0.1146, 0.0352)	21974
	Quartile 3	-0.0004	0.9927	(-0.0797, 0.079)	21974
	Quartile 4	-0.0054	0.8984	(-0.0879, 0.0771)	21974
CO	Linear	0.0154	<0.0001*	(0.0081, 0.0227)	24260
	Log linear	0.0053	0.4161	(-0.0074, 0.0179)	24275
	Categorical [Ref. Quartile 1]				
	Quartile 2	-0.0311	0.3833	(-0.1009, 0.0388)	24276
	Quartile 3	-0.0643	0.0812	(-0.1367, 0.008)	24276
	Quartile 4	0.0495	0.1913	(-0.0247, 0.1237)	24276

Note: All exposure-response models controlled for maternal age, mother's highest education level, BMI at baseline, household food insecurity, mother's diet diversity, exposure to secondhand smoke, gestational age at the hemoglobin measurement, gestational age squared and IRC.

Table S19. Exposure-response analysis results from mixed-effects models – Guatemala

Exposures	Model Type	Estimate	p-value	95% CI	AIC
Crude Associations					
$PM_{2.5}$	Linear	0.0007	0.0006	(0.0003, 0.0011)	5977
	Log linear	0.1030	0.0000	(0.0584, 0.1476)	5959
	Categorical [Ref. Quartile 1]				
	Quartile 2	0.1027	0.0976	(-0.0188, 0.2242)	5973
	Quartile 3	0.1555	0.0145	(0.0309, 0.2801)	5973
	Quartile 4	0.2641	0.0000	(0.1377, 0.3905)	5973
BC	Linear	0.0043	0.1161	(-0.0011, 0.0098)	5774
	Log linear	0.1277	0.0001	(0.0635, 0.1919)	5756
	Categorical [Ref. Quartile 1]				
	Quartile 2	0.0704	0.2707	(-0.0548, 0.1956)	5762
	Quartile 3	0.2570	0.0001	(0.1303, 0.3838)	5762
	Quartile 4	0.2155	0.0010	(0.0872, 0.3438)	5762
CO	Linear	0.0111	0.2255	(-0.0068, 0.029)	6112
	Log linear	0.0163	0.1451	(-0.0056, 0.0382)	6111
	Categorical [Ref. Quartile 1]				
	Quartile 2	0.0000	0.9998	(-0.1167, 0.1167)	6120
	Quartile 3	0.0527	0.3819	(-0.0654, 0.1708)	6120
	Quartile 4	0.0507	0.4119	(-0.0703, 0.1716)	6120
Adjusted Associations					
$PM_{2.5}$	Linear	0.0001	0.5857	(-0.0003, 0.0005)	5798
	Log linear	0.0051	0.8310	(-0.0421, 0.0524)	5788
	Categorical [Ref. Quartile 1]				
	Quartile 2	-0.0189	0.7505	(-0.1354, 0.0976)	5798
	Quartile 3	-0.0510	0.4240	(-0.1762, 0.0741)	5798
	Quartile 4	0.0046	0.9455	(-0.1272, 0.1364)	5798
BC	Linear	-0.0029	0.2814	(-0.0082, 0.0024)	5595
	Log linear	-0.0122	0.7220	(-0.0792, 0.0549)	5591
	Categorical [Ref. Quartile 1]				
	Quartile 2	-0.0712	0.2488	(-0.1921, 0.0497)	5599
	Quartile 3	0.0111	0.8654	(-0.1174, 0.1396)	5599
	Quartile 4	-0.0424	0.5294	(-0.1744, 0.0897)	5599
CO	Linear	-0.0065	0.4598	(-0.0237, 0.0107)	5925
	Log linear	-0.0176	0.1132	(-0.0394, 0.0042)	5922
	Categorical [Ref. Quartile 1]				
	Quartile 2	-0.0782	0.1660	(-0.1889, 0.0324)	5929
	Quartile 3	-0.1118	0.0577	(-0.2273, 0.0036)	5929
	Quartile 4	-0.1159	0.0569	(-0.235, 0.0033)	5929

Note: All exposure-response models controlled for maternal age, mother's highest education level, BMI at baseline, household food insecurity, mother's diet diversity, exposure to secondhand smoke, gestational age at the hemoglobin measurement and gestational age squared.

Table S20. Exposure-response analysis results from mixed-effects models – India

Exposures	Model Type	Estimate	p-value	95% CI	AIC
Crude Associations					
$PM_{2.5}$	Linear	-0.0002	0.3497	(-0.0007, 0.0002)	6085
	Log linear	-0.0074	0.8089	(-0.0672, 0.0525)	6076
	Categorical [Ref. Quartile 1]				
	Quartile 2	-0.0618	0.4128	(-0.2096, 0.086)	6084
	Quartile 3	0.0130	0.8651	(-0.1373, 0.1633)	6084
	Quartile 4	-0.0589	0.4527	(-0.2126, 0.0948)	6084
BC	Linear	0.0018	0.5010	(-0.0034, 0.007)	5951
	Log linear	0.0194	0.4698	(-0.0332, 0.072)	5946
	Categorical [Ref. Quartile 1]				
	Quartile 2	-0.0362	0.6393	(-0.1876, 0.1152)	5953
	Quartile 3	0.0540	0.4903	(-0.0994, 0.2075)	5953
	Quartile 4	0.0593	0.4545	(-0.096, 0.2145)	5953
CO	Linear	0.0122	0.1770	(-0.0055, 0.0299)	6544
	Log linear	0.0055	0.5886	(-0.0143, 0.0252)	6545
	Categorical [Ref. Quartile 1]				
	Quartile 2	0.0531	0.4604	(-0.0878, 0.1939)	6552
	Quartile 3	0.0422	0.5638	(-0.101, 0.1853)	6552
	Quartile 4	0.0448	0.5404	(-0.0986, 0.1883)	6552
Adjusted Associations					
$PM_{2.5}$	Linear	-0.0002	0.4111	(-0.0007, 0.0003)	6114
	Log linear	-0.0053	0.8693	(-0.0685, 0.0579)	6104
	Categorical [Ref. Quartile 1]				
	Quartile 2	-0.0704	0.3519	(-0.2187, 0.0778)	6112
	Quartile 3	-0.0023	0.9771	(-0.1582, 0.1536)	6112
	Quartile 4	-0.0654	0.4251	(-0.2261, 0.0953)	6112
BC	Linear	0.0014	0.6159	(-0.004, 0.0068)	5980
	Log linear	0.0136	0.6389	(-0.0433, 0.0706)	5975
	Categorical [Ref. Quartile 1]				
	Quartile 2	-0.0667	0.3920	(-0.2193, 0.086)	5982
	Quartile 3	0.0370	0.6537	(-0.1248, 0.1989)	5982
	Quartile 4	0.0496	0.5585	(-0.1164, 0.2155)	5982
CO	Linear	0.0101	0.2583	(-0.0074, 0.0277)	6560
	Log linear	0.0018	0.8664	(-0.0188, 0.0223)	6561
	Categorical [Ref. Quartile 1]				
	Quartile 2	0.0301	0.6761	(-0.111, 0.1712)	6568
	Quartile 3	0.0251	0.7381	(-0.1219, 0.172)	6568
	Quartile 4	0.0167	0.8238	(-0.1305, 0.164)	6568

Note: All exposure-response models controlled for maternal age, mother's highest education level, BMI at baseline, household food insecurity, mother's diet diversity, exposure to secondhand smoke, gestational age at the hemoglobin measurement and gestational age squared.

Table S21. Exposure-response analysis results from mixed-effects models – Peru

Exposures	Model Type	Estimate	p-value	95% CI	AIC
Crude Associations					
$PM_{2.5}$	Linear	0.0011	0.0001	(0.0006, 0.0017)	5220
	Log linear	0.1757	0.0000	(0.1185, 0.2329)	5191
	Categorical [Ref. Quartile 1]				
	Quartile 2	0.0441	0.5759	(-0.1104, 0.1986)	5202
	Quartile 3	0.2979	0.0002	(0.1434, 0.4525)	5202
	Quartile 4	0.3961	0.0000	(0.2379, 0.5542)	5202
BC	Linear	0.0176	0.0000	(0.0118, 0.0233)	4758
	Log linear	0.2061	0.0000	(0.1507, 0.2615)	4737
	Categorical [Ref. Quartile 1]				
	Quartile 2	0.0870	0.2881	(-0.0735, 0.2475)	4756
	Quartile 3	0.3734	0.0000	(0.213, 0.5337)	4756
	Quartile 4	0.4584	0.0000	(0.2936, 0.6232)	4756
CO	Linear	0.0259	0.0000	(0.015, 0.0368)	5105
	Log linear	0.0700	0.0000	(0.0407, 0.0993)	5103
	Categorical [Ref. Quartile 1]				
	Quartile 2	0.0922	0.2538	(-0.0661, 0.2506)	5109
	Quartile 3	0.0718	0.3808	(-0.0887, 0.2323)	5109
	Quartile 4	0.3756	0.0000	(0.2127, 0.5385)	5109
Adjusted Associations					
$PM_{2.5}$	Linear	0.0003	0.2250	(-0.0002, 0.0009)	4952
	Log linear	0.0266	0.3613	(-0.0305, 0.0838)	4943
	Categorical [Ref. Quartile 1]				
	Quartile 2	-0.0238	0.7435	(-0.1666, 0.1189)	4952
	Quartile 3	0.0692	0.3549	(-0.0773, 0.2157)	4952
	Quartile 4	0.0057	0.9434	(-0.1518, 0.1632)	4952
BC	Linear	0.0057	0.0464	(0.0001, 0.0114)	4537
	Log linear	0.0444	0.1333	(-0.0135, 0.1023)	4534
	Categorical [Ref. Quartile 1]				
	Quartile 2	0.0482	0.5255	(-0.1006, 0.197)	4544
	Quartile 3	0.0628	0.4286	(-0.0926, 0.2182)	4544
	Quartile 4	0.0494	0.5609	(-0.117, 0.2157)	4544
CO	Linear	0.0163	0.0018	(0.0061, 0.0265)	4881
	Log linear	0.0283	0.0501	(0, 0.0567)	4884
	Categorical [Ref. Quartile 1]				
	Quartile 2	0.0018	0.9814	(-0.146, 0.1496)	4886
	Quartile 3	-0.0884	0.2530	(-0.24, 0.0631)	4886
	Quartile 4	0.1617	0.0433	(0.005, 0.3185)	4886

Note: All exposure-response models controlled for maternal age, mother's highest education level, BMI at baseline, household food insecurity, mother's diet diversity, exposure to secondhand smoke, gestational age at the hemoglobin measurement and gestational age squared.

Table S22. Exposure-response analysis results from mixed-effects models – Rwanda

Exposures	Model Type	Estimate	p-value	95% CI	AIC
Crude Associations					
$PM_{2.5}$	Linear	0.0000	0.8886	(-0.0006, 0.0007)	6630
	Log linear	0.0286	0.4800	(-0.0508, 0.108)	6620
	Categorical [Ref. Quartile 1]				
	Quartile 2	0.0602	0.4774	(-0.1058, 0.2262)	6629
	Quartile 3	0.0429	0.6196	(-0.1263, 0.212)	6629
	Quartile 4	0.0799	0.3688	(-0.0943, 0.2542)	6629
BC	Linear	0.0042	0.2985	(-0.0037, 0.012)	5875
	Log linear	0.0541	0.2827	(-0.0445, 0.1527)	5870
	Categorical [Ref. Quartile 1]				
	Quartile 2	0.0905	0.3232	(-0.089, 0.2699)	5879
	Quartile 3	0.0426	0.6515	(-0.1423, 0.2275)	5879
	Quartile 4	0.0881	0.3628	(-0.1015, 0.2777)	5879
CO	Linear	0.0367	0.0000	(0.019, 0.0544)	6907
	Log linear	0.0718	0.0000	(0.038, 0.1056)	6905
	Categorical [Ref. Quartile 1]				
	Quartile 2	0.0736	0.3636	(-0.0852, 0.2324)	6910
	Quartile 3	0.0851	0.3043	(-0.0773, 0.2475)	6910
	Quartile 4	0.3569	0.0000	(0.1915, 0.5224)	6910
Adjusted Associations					
$PM_{2.5}$	Linear	-0.0003	0.3361	(-0.001, 0.0003)	6544
	Log linear	-0.0442	0.2988	(-0.1275, 0.0391)	6534
	Categorical [Ref. Quartile 1]				
	Quartile 2	0.0199	0.8106	(-0.1427, 0.1825)	6542
	Quartile 3	-0.1003	0.2522	(-0.2718, 0.0713)	6542
	Quartile 4	-0.0766	0.4096	(-0.2586, 0.1054)	6542
BC	Linear	0.0006	0.8763	(-0.0073, 0.0085)	5815
	Log linear	-0.0323	0.5423	(-0.1364, 0.0717)	5810
	Categorical [Ref. Quartile 1]				
	Quartile 2	0.0203	0.8212	(-0.156, 0.1967)	5817
	Quartile 3	-0.1090	0.2584	(-0.2978, 0.0799)	5817
	Quartile 4	-0.0746	0.4598	(-0.2724, 0.1232)	5817
CO	Linear	0.0229	0.0104	(0.0054, 0.0403)	6828
	Log linear	0.0370	0.0356	(0.0025, 0.0714)	6829
	Categorical [Ref. Quartile 1]				
	Quartile 2	-0.0218	0.7861	(-0.1793, 0.1357)	6831
	Quartile 3	-0.0329	0.6910	(-0.1949, 0.1292)	6831
	Quartile 4	0.1945	0.0230	(0.027, 0.362)	6831

Note: All exposure-response models controlled for maternal age, mother's highest education level, BMI at baseline, household food insecurity, mother's diet diversity, exposure to secondhand smoke, gestational age at the hemoglobin measurement and gestational

Table S23. Exploratory analysis results of ITT effects among women who were anemic at baseline – all IRCs and by country

Hemoglobin concentration (continuous)	N	Estimate (g/dL)	95% CI	p-value
Pooled (all participants)	1832	0.091	(-0.044, 0.225)	0.187
Guatemala	162	0.076	(-0.376, 0.527)	0.743
India	933	0.155	(-0.029, 0.339)	0.1
Peru	443	-0.017	(-0.269, 0.234)	0.894
Rwanda	294	0.114	(-0.27, 0.499)	0.56
