# Supplementary material for: Effects of cooking with liquefied petroleum gas versus biomass on hemoglobin concentrations in pregnant women: a pre-specified exploratory analysis of the HAPIN trial
Source: Nat Commun. 2026 Jun 10;17:7393. doi: 10.1038/s41467-026-74114-9 (PMC13402313; doi:10.1038/s41467-026-74114-9)
Supplement: Supplementary file 2 — Reporting summary [file 41467_2026_74114_MOESM2_ESM.pdf]

nature portfolio

Corresponding author(s): Sheela S. Sinharoy

Last updated by author(s): Aug 16, 2025

Reporting Summary

Nature Portfolio wishes to improve the reproducibility of the work that we publish. This form provides structure for consistency and transparency in reporting. For further information on Nature Portfolio policies, see our Editorial Policies and the Editorial Policy Checklist.

Statistics

For all statistical analyses, confirm that the following items are present in the figure legend, table legend, main text, or Methods section.

n/a	Confirmed	
☐	☒	The exact sample size (*n*) for each experimental group/condition, given as a discrete number and unit of measurement
☐	☒	A statement on whether measurements were taken from distinct samples or whether the same sample was measured repeatedly
☐	☒	The statistical test(s) used AND whether they are one- or two-sided<br>*Only common tests should be described solely by name; describe more complex techniques in the Methods section.*
☐	☒	A description of all covariates tested
☐	☒	A description of any assumptions or corrections, such as tests of normality and adjustment for multiple comparisons
☐	☒	A full description of the statistical parameters including central tendency (e.g. means) or other basic estimates (e.g. regression coefficient) AND variation (e.g. standard deviation) or associated estimates of uncertainty (e.g. confidence intervals)
☐	☒	For null hypothesis testing, the test statistic (e.g. *F*, *t*, *r*) with confidence intervals, effect sizes, degrees of freedom and *P* value noted<br>*Give P values as exact values whenever suitable.*
☒	☐	For Bayesian analysis, information on the choice of priors and Markov chain Monte Carlo settings
☒	☐	For hierarchical and complex designs, identification of the appropriate level for tests and full reporting of outcomes
☒	☐	Estimates of effect sizes (e.g. Cohen's *d*, Pearson's *r*), indicating how they were calculated

Our web collection on statistics for biologists contains articles on many of the points above.

Software and code

Policy information about availability of computer code

Data collection	We used the Research Electronic Data Capture (REDCap) mobile application to collect the data in this study. There is no version number. We have cited a paper that describes this study's use of REDCap in detail.
Data analysis	We used R version 4.2.2 to conduct all analyses in this study. For statistical modeling, we used the R package lmerTest Version [1] '3.1.3'.

For manuscripts utilizing custom algorithms or software that are central to the research but not yet described in published literature, software must be made available to editors and reviewers. We strongly encourage code deposition in a community repository (e.g. GitHub). See the Nature Portfolio guidelines for submitting code & software for further information.

Data

Policy information about availability of data

All manuscripts must include a data availability statement. This statement should provide the following information, where applicable:

- Accession codes, unique identifiers, or web links for publicly available datasets
- A description of any restrictions on data availability
- For clinical datasets or third party data, please ensure that the statement adheres to our policy

The anonymized data underlying the findings of this study and accompanying data dictionary will be made publicly available upon publication of the manuscript via the Emory Dataverse online data repository: https://doi.org/10.15139/S3/M4N5QO

Research involving human participants, their data, or biological material

Policy information about studies with human participants or human data. See also policy information about sex, gender (identity/presentation), and sexual orientation and race, ethnicity and racism.

Reporting on sex and gender	The target population in HAPIN was pregnant women, enrolled during pregnancy, hence all samples are from females. This is appropriate given the HAPIN trial's primary aims related to birth outcomes and also given women's primary responsibility for cooking, resulting in higher exposure to household air pollution, and greater risk of anemia due to higher biological requirements for iron in pregnancy.
Reporting on race, ethnicity, or other socially relevant groupings	We do not use race, ethnicity, or similar categorical variables in our analysis. We use geographic study location (Guatemala, Peru, India, Rwanda) but do not further categorize participants within geographies by ethnicity.<br><br>In addition to geographic location, we include the following covariates in some of our analyses, as specified in the manuscript: maternal age, maternal highest level of education completed, maternal body mass index at baseline, household food insecurity, maternal diet diversity, and exposure to secondhand smoke.
Population characteristics	This information is presented in the manuscript (see Table 1). Women were between 18-35 years of age; the majority of women had not completed secondary school; mean body mass index at baseline was 23; most households were food secure; most women had low dietary diversity; and exposure to secondhand smoke was minimal.
Recruitment	We identified and recruited women through antenatal clinics in each geographic location. Women were recruited by staff within the clinics when they came to the clinics for antenatal care and by community health workers. While antenatal clinic attendance is high in all four of our study settings, selection bias is possible if women who did not obtain antenatal care and who did not engage with community health workers were systematically missed from our recruitment. The potential impact of selection bias on our results is likely to be minimal. Self-selection and other forms of bias are unlikely.
Ethics oversight	This information is presented in the manuscript. The study protocol was reviewed and approved by institutional review boards or ethics committees at Emory University (00089799), Johns Hopkins University (00007403), Sri Ramachandra Institute of Higher Education and Research (IEC-N1/16/JUL/54/49), the Indian Council of Medical Research – Health Ministry Screening Committee (5/8/4-30/(Env)/Indo-US/2016-NCD-I), Universidad del Valle de Guatemala (146-08-2016/11-2016), Guatemalan Ministry of Health National Ethics Committee (11-2016), A.B. PRISMA (CE3571·16), the London School of Hygiene and Tropical Medicine (11664-5), the Rwandan National Ethics Committee (No.357/RNEC/2018), and Washington University in St. Louis (201611159).

Note that full information on the approval of the study protocol must also be provided in the manuscript.

Field-specific reporting

Please select the one below that is the best fit for your research. If you are not sure, read the appropriate sections before making your selection.

☐ Life sciences ☒ Behavioural & social sciences ☐ Ecological, evolutionary & environmental sciences

For a reference copy of the document with all sections, see nature.com/documents/nr-reporting-summary-flat.pdf

Behavioural & social sciences study design

All studies must disclose on these points even when the disclosure is negative.

Study description	Study type: randomized control trial<br>Data type: quantitative
Research sample	The HAPIN study sample was pregnant women ages 18-35 in India, Peru, Guatemala and Rwanda. The study sample was not intended to be representative at the national level. The rationale for the study sample is due to the HAPIN trial's primary aims related to birth outcomes and also given women's primary responsibility for cooking, resulting in higher exposure to household air pollution, and greater risk of anemia due to higher biological requirements for iron in pregnancy.
Sampling strategy	The sampling procedure was a stratified sample of women who were recruited through antenatal clinics and by community health workers across ten geographic strata in four countries. The sample size was calculated based on the primary outcomes of the HAPIN trial. Specifically, for each of the four primary outcomes, we calculated sample size and minimal detectable effect associated with an 80% power and a type I error rate a level of 0.0125, assuming a 10% attrition during follow-up. Our calculations utilized baseline prevalence for stunting (30%), incidence for childhood pneumonia (0.26 cases per child-year), and standard deviations for birth weight (178 g) and blood pressure (12 mmHg) from published results in other LMIC settings.
Data collection	Data were collected in person at the household level by trained staff. Data on socio-demographic characteristics, medical history, and cooking and other behavioral practices were collected from pregnant women using structured survey modules that were programmed on tablets equipped with the Research Electronic Data Capture (REDCap) mobile application. Hemoglobin concentration was measured from a single drop of capillary blood obtained via finger prick, using the HemoCue® Hb 201+ System. Due to the nature of the intervention, it was not possible to mask participants or data collection teams to the group assignment. However, the study investigators were blinded to the collected data.

Timing	Start date: 07 May 2018<br>Stop date: 24 March 2020
Data exclusions	Participants were excluded (N=6) from the statistical analysis if they reported any current smoking. This exclusion was in line with the pre-established trial exclusion criteria (i.e., these participants should not have been enrolled in the trial). In addition, participants were excluded (N=11) for having baseline hemoglobin concentrations <7.0 g/dL at baseline. This exclusion criterion was established during the development of the pre-specified statistical analysis plan because we had intervened on these participants by referring them for additional management of their anemia.
Non-participation	A total of 94 participants (2.9%) dropped out of the study, for the following reasons: voluntary withdrawal from the study (N=31), withdrawal by study team (N=4), moved out of study area (N=17), or pregnancy loss (N=42).
Randomization	Participants were randomly assigned in a 1:1 ratio stratified by setting (ten geographic strata, as listed in the manuscript) in permuted blocks of two and four to either receive the intervention or continue their traditional cooking practices with biomass fuels.

Reporting for specific materials, systems and methods

We require information from authors about some types of materials, experimental systems and methods used in many studies. Here, indicate whether each material, system or method listed is relevant to your study. If you are not sure if a list item applies to your research, read the appropriate section before selecting a response.

Materials & experimental systems

n/a	Involved in the study
☒	☐ Antibodies
☒	☐ Eukaryotic cell lines
☒	☐ Palaeontology and archaeology
☒	☐ Animals and other organisms
☐	☒ Clinical data
☒	☐ Dual use research of concern
☒	☐ Plants

Methods

n/a	Involved in the study
☒	☐ ChIP-seq
☒	☐ Flow cytometry
☒	☐ MRI-based neuroimaging

Clinical data

Policy information about clinical studies

All manuscripts should comply with the ICMJE guidelines for publication of clinical research and a completed CONSORT checklist must be included with all submissions.

Clinical trial registration	NCT02944682
Study protocol	https://clinicaltrials.gov/study/NCT02944682?term=NCT02944682
Data collection	We enrolled and randomized 3200 pregnant women from India, Peru, Guatemala and Rwanda between May 7, 2018, and February 29, 2020. Collection of data presented in this manuscript took place during household visits at baseline and then at two follow-up time points (24-28 and 32-36 weeks gestation). The last day of data collection for the second follow-up time point (32-36 weeks gestation) was August 25, 2020.
Outcomes	Primary and secondary outcomes were pre-defined based on evidence gaps as part of the proposal writing process and are listed (along with methods for their assessment) in the clinical trial registration (see URL above). Primary outcomes (stunting, pneumonia, birth weight, and blood pressure) are assessed using standard methods for measurement of anthropometry (stunting / birth weight) and following standard clinical guidelines (pneumonia / blood pressure). The secondary outcome presented here, hemoglobin concentration, is assessed from a single drop of capillary blood obtained via finger prick, using the HemoCue® Hb 201+ System.

Plants

Seed stocks	*Report on the source of all seed stocks or other plant material used. If applicable, state the seed stock centre and catalogue number. If plant specimens were collected from the field, describe the collection location, date and sampling procedures.*
Novel plant genotypes	*Describe the methods by which all novel plant genotypes were produced. This includes those generated by transgenic approaches, gene editing, chemical/radiation-based mutagenesis and hybridization. For transgenic lines, describe the transformation method, the number of independent lines analyzed and the generation upon which experiments were performed. For gene-edited lines, describe the editor used, the endogenous sequence targeted for editing, the targeting guide RNA sequence (if applicable) and how the editor was applied.*
Authentication	*Describe any authentication procedures for each seed stock used or novel genotype generated. Describe any experiments used to assess the effect of a mutation and, where applicable, how potential secondary effects (e.g. second site T-DNA insertions, mosiacism, off-target gene editing) were examined.*
