# Supplementary material for: Effects of cooking with liquefied petroleum gas versus biomass on hemoglobin concentrations in pregnant women: a pre-specified exploratory analysis of the HAPIN trial
Source: Nat Commun. 2026 Jun 10;17:7393. doi: 10.1038/s41467-026-74114-9 (PMC13402313; doi:10.1038/s41467-026-74114-9)
Supplement: Supplementary file 3 — Transparent Peer Review file [file 41467_2026_74114_MOESM3_ESM.pdf]

nature portfolio

Peer Review File

Effects of cooking with liquefied petroleum gas versus biomass on hemoglobin concentrations in pregnant women: a pre-specified exploratory analysis of the HAPIN trial

Corresponding Author: Dr Sheela Sinharoy

This file contains all reviewer reports in order by version, followed by all author rebuttals in order by version.

Version 0:

Reviewer comments:

Reviewer #1

(Remarks to the Author)
The authors present a well written description of HAPIN, a large multi-country household air pollution intervention trial that has numerous strengths. The outcome analyzed for this manuscript, anemia and hemoglobin concentration, is among a large list of tertiary ('other') outcomes listed in the protocol and trial registration, and the authors are very upfront about this context. Despite being a lower-tier outcome, it is important that these and other analyses are presented in order to realize the full value of this large, well-designed study. That said, the authors might be over-playing the significance of the findings presented here. The reviewer offers here some further considerations for placing these findings in the proper context of the current literature. None of these considerations should be characterized as requiring major revision.

The ITT findings and the exposure-response findings appear to be in opposing directions. The ITT results are in the a priori hypothesized direction, greater hemoglobin concentration among women in the pollution-reducing LPG intervention compared to higher biomass combustion exposure control group. However, the readers would benefit from a simpler characterization of the clinical significance of the observed effect estimate. Based on the baseline measures of hemoglobin concentration (11.4-11.5 mg/dL), the intervention effect might translate to a 0.6% change. In the discussion the authors do characterize the ITT effect as small without corroborating impact on the anemia findings, but causal readers of the abstract and results sections could be misled.

The pollutant-specific exposure-response results run counter to the ITT fundings, specifically the CO results. CO exposure is found to be associated with greater hemoglobin concentrations. Limited prior literature suggests that air pollution generally and CO exposure specifically are associated with lower hemoglobin concentrations (Honda 2017, PMC5361751; Hwang 2024, PMC11003135). As population-based studies evaluating ambient exposures, these studies are clearly different from HAPIN. Nevertheless, it is difficult reconcile these studies with the current HAPIN findings. Given the prior epidemiology, the inconsistent findings across HAPIN sites, and the small effect (perhaps 0.1% change per unit of CO) the authors may be stretching when they suggest "chronic CO poisoning." Perhaps this language could be supported with some health-based criteria that are in-line with these CO-based changes in hemoglobin concentration?

Some additional comments for the authors to consider:

Is it possible that the exposure-response CO linear effect is heavily driven by a strong effect in the highest exposed? The effect estimates by exposure quartile do not suggest a monotonic dose response relations, but the effects are large, in relative terms, for Q4 (Table S18). This reviewer is uncertain what the next analysis step might be to further explore this question – a threshold effect? Is it possible that further evaluation of a threshold could reveal more clinically-relevant findings among the highest exposure group?

Reviewer #2

(Remarks to the Author)
This is an interesting study.

Methods
Lines 142-143. “We sought to randomly assign 1600 participants to the intervention arm and 1600 to the control arm.”. How was the sample size determined?
In the statistical analysis section, it is mentioned that “We conducted an intention-to-treat (ITT) analysis to assess the effect of the LPG cookstove and fuel intervention on hemoglobin in pregnant women using a mixed-effects model that takes into account the non-independence of the hemoglobin concentration at each time point.”. However, the time variable was not included in the analysis shown in Table 3. Please clarify.
Lines 258-260. “we included variables in the models if they were hypothesized to be predictors of the outcome to decrease the amount of unexplained variability in the data and increase power to estimate an association.”. It’s not clear how the amount of unexplained variability and power were assessed.
Lines 266-267. “we first fit linear models with different exposure forms (i.e., linear and log-linear)”. Please explain why log-linear was used.
Line 276. “All analyses were performed using R version 4.2.2”. Please provide all packages used in the analysis.

Results
Table 1. What dose “*” mean?
Table 3. Suggest changing a mixed-effect model as a linear mixed-effect model. Noted that in the results of “0.074 g/dL (95% CI: 0.002, 0.145)”, it looks that the 95%CI is very wide, which may indicate the uncertainty in the estimate. This should be discussed in the discussion section.
Table 4. Noted that the linear or log linear was used. Suggest presenting the backtransformed values and backtransformed 95% CI if log-transformation was used. In addition, how were the missing values handled in the analysis?

Reviewer #3

(Remarks to the Author)
This is an important study of secondary analyses from the large multi-country Household Air Pollution Intervention Network (HAPIN) study. The main randomized controlled trial (RCT) compared liquefied petroleum gas (LPG) fuel and cookstove use to ongoing use of biomass fuels and traditional cookstoves by pregnant women, and multiple primary and secondary results have been published.

The general lack of impact of the intervention on anemia rates but positive correlation with hemoglobin concentrations are important findings, as are the findings of an association of CO with a small increase in hemoglobin concentrations. I was surprised to see no mention of standard of care and iron (of iron folate; IFA) intake in the study population. One assumes that barring severe anemia cases which were excluded, that study pregnant women continued to receive standard antenatal care including IFA. What was the intake in both groups? Was any difference noted by compliance? Did these women receive any other supplements especially as many had a BMI<18.5?

Although most women were recruited well after the first trimester (mean gestational age around 15 weeks), were any differences found by length of exposure (those recruited before 14-16 weeks gestation versus late presenters)?

It would have been useful to show maternal morbidities and birth outcomes by anemia subgroups (moderate, mild or non-anemic women).

Reviewer #4

(Remarks to the Author)
Abstract:
1, any clinical consideration for the three timepoints?
2, please check the two values (0.074 g/dL, 95% CI: 0.002, 0.145) vs 0.015 g/dL (95% CI: 0.002, 0.145).
3, Since the CO is part of the air quality, it is very hard to have an intervention aiming on this.
4, multiplicity would be expected after multiply comparisons. Chace findings would not excluded, particularly for CO.
5, I would recommend stating that further studies are needed to validate this finings rather than focusing on the interven on CO.
Introduction
Looks fines
Method.
1,This analysis is based on a published study with primary outcomes on low birth weight, severe pneumonia, and stunting in infants, along with high blood pressure in non-pregnant adult women residing in the household. The outcome chosen in this study is a secondary outcome. proper mutilplties needs to be took in account. Otherwise, clearly statement about the nature of exploratory analysis is needed to remind the readers.
2, “The HAPIN Data Management Core generated randomization lists based on block randomization using randomly selected block sizes, then prepared individual sealed envelopes containing trial group allocation, which were shipped to each study site and selected by participants” what do you mean selected by participants???
3, is there analysis plan for this analysis (not this trial)? ITT defined in this analysis did not follow the standard. Why do smokers exclude?
4, there is no need to describe the formulations with decription is the main text. I would suggest moving this in the appendix or refer to the SAP.

5, Please define the minimal clinical relevant margin in the method.
Result:
1, a lot of P values in this section without alpha adjustment.
2, any justification for the exclusion of baseline hemoglobin concentrations <7.0 g/dL at baseline?
3, what is the definition of anemia?? the proportion of anemia differ so significantly? Any reason? What is the implication of this group unbalance.
4, please provide the number of subjects for the analysis in Table S23

Discussion
1, Please explain the reasons of having different findings for the anemia and Hb. Currently, the study focus on the HB value. I suspect the outcome reporting bias.
2, Positive results related to CO may be a chance finding. How would an intervene would be target the CO
3, discussion around the clinical relevance and the statisticant significance may be helpful to the readers. The current version mainly place their focus on the statiscant significance.

Version 1:

Reviewer comments:

Reviewer #1

(Remarks to the Author)
The authors have adequately responded to prior comments and made changes to the manuscript, where appropriate.

Reviewer #2

(Remarks to the Author)
The authors have responded well to my questions. The manuscript is much clearer now.

Reviewer #3

(Remarks to the Author)
I think the authors have responded reasonably well to the critique and comments. In terms of the analysis of impact by anemia categories, it is a natural question readers may ask and including a sentence as to why this isn't part of the current analysis, or not feasible, would be important. Providing some of that prevalence data from the baseline would also allow a comparative evaluation of the population in reference to other geographies.

Reviewer #4

(Remarks to the Author)
The authors have satisfactorily addressed my previous concerns. I have one further comment regarding the clinical relevance of the findings in light of the small effect size. I recommend that this point be explicitly highlighted in the Discussion section to ensure readers are aware of its implications.

Open Access This Peer Review File is licensed under a Creative Commons Attribution 4.0 International License, which permits use, sharing, adaptation, distribution and reproduction in any medium or format, as long as you give appropriate credit to the original author(s) and the source, provide a link to the Creative Commons license, and indicate if changes were made.
In cases where reviewers are anonymous, credit should be given to 'Anonymous Referee' and the source.
The images or other third party material in this Peer Review File are included in the article’s Creative Commons license, unless indicated otherwise in a credit line to the material. If material is not included in the article’s Creative Commons license and your intended use is not permitted by statutory regulation or exceeds the permitted use, you will need to obtain permission directly from the copyright holder.
To view a copy of this license, visit https://creativecommons.org/licenses/by/4.0/

Response to reviewers

Reviewer Comment	Authors' Response	Changes made to manuscript: underline indicates new text; strikethrough indicates deleted text
**Reviewer #1 (Remarks to the Author):**		
The authors present a well written description of HAPIN, a large multi-country household air pollution intervention trial that has numerous strengths. The outcome analyzed for this manuscript, anemia and hemoglobin concentration, is among a large list of tertiary ('other') outcomes listed in the protocol and trial registration, and the authors are very upfront about this context. Despite being a lower-tier outcome, it is important that these and other analyses are presented in order to realize the full value of this large, well-designed study. That said, the authors might be over-playing the significance of the findings presented here. The reviewer offers here some further considerations for placing these findings in the proper context of the current literature. None of these considerations should be characterized as requiring major revision.	**Response:** Thank you for this fair and balanced assessment of our work. We appreciate the reviewer's feedback and have responded to each consideration below.	
The ITT findings and the exposure-response findings appear to be in opposing directions. The ITT results are in the a priori hypothesized direction, greater hemoglobin concentration among women in the pollution-reducing LPG intervention compared to higher biomass combustion exposure control group. However, the readers would benefit from a simpler characterization of the clinical significance of the observed effect estimate. Based on the baseline measures of hemoglobin concentration (11.4-11.5 mg/dL), the intervention effect might translate to a	**Response:** We have revised the first paragraph of the Discussion section and added a new paragraph immediately after that one to address this concern. In the new paragraph, we interpret the clinical significance of the observed effect estimate and place our results in context of the larger evidence base related to interventions to increase hemoglobin concentrations among pregnant women.	The effect size, while statistically significant, was small<u> and likely not clinically meaningful at the individual level.</u><br><br><u>The effect size observed in our study (0.074 g/dL) is small, especially compared to interventions designed to increase iron status through direct approaches for an individual person or patient. For example, the standard of care for pregnant women</u>

0.6% change. In the discussion the authors do characterize the ITT effect as small without corroborating impact on the anemia findings, but causal readers of the abstract and results sections could be misled.		is daily oral iron and folic acid (IFA) supplementation.[32] A recent systematic review and meta-analysis found that daily oral iron supplementation had a mean effect size on hemoglobin concentration among pregnant women at or near term (≥34 weeks gestation) of 0.953 g/dL (95% CI: 0.699, 1.206).[33] RCTs of other interventions to increase iron intake among pregnant women through dietary supplementation have had mixed results: iron-fortified beverages were found to increase Hb concentrations (0.416 g/dL) in Tanzania but not in Vietnam, while daily food supplementation was found to have no effect on Hb concentration in Indonesia.[34-36] Another RCT of iron-fortified food supplementation among pregnant women in Cambodia did not report Hb concentrations but reported a significant protective effect on anemia (Odds Ratio=0.51; 95% CI: 0.34, 0.77).[37] The observed effect size in our trial, while not clinically meaningful alone, is larger than has been observed in some dietary interventions and could complement the standard of care IFA supplementation as part of a larger public health, population-level anemia prevention and control strategy.
The pollutant-specific exposure-response results run counter to the ITT fundings, specifically the CO results. CO exposure is found to be associated with greater hemoglobin concentrations. Limited prior literature	**Response:** We have added text to the **Introduction** to clarify that CO exposures should be expected to have a positive association with hemoglobin concentration,	**Introduction:** At the same time, CO may have the opposite effect, as it is known to bind with hemoglobin to form carboxyhemoglobin, leading to hypoxia

suggests that air pollution generally and CO exposure specifically are associated with lower hemoglobin concentrations (Honda 2017, PMC5361751; Hwang 2024, PMC11003135). As population-based studies evaluating ambient exposures, these studies are clearly different from HAPIN. Nevertheless, it is difficult reconcile these studies with the current HAPIN findings. Given the prior epidemiology, the inconsistent findings across HAPIN sites, and the small effect (perhaps 0.1% change per unit of CO) the authors may be stretching when they suggest "chronic CO poisoning." Perhaps this language could be supported with some health-based criteria that are in-line with these CO-based changes in hemoglobin concentration?	not a negative association as documented by Hwang and Kim 2024. (In the Honda et al. 2017 paper, the exposure assessment includes only $PM_{2.5}$ and NO_2, not CO.)<br><br>The reviewer's point about our text related to "chronic CO poisoning" is fair. We have removed this wording from the **Abstract** and the **Discussion** section of the manuscript.	and, hence, increased erythropoiesis and increased hemoglobin production.[7] Reductions in carbon monoxide exposure may therefore lead to physiologic reductions in hemoglobin and, concurrently, an apparent increase in prevalence of anemia.<br><br>**Abstract:** ~~Our finding of positive associations between hemoglobin concentration and CO exposure may represent functional evidence of chronic, population-level CO poisoning~~ Further research may be needed to examine the biological mechanisms underlying our findings.<br><br>**Discussion:** ~~The higher hemoglobin levels associated with higher CO exposure in our study are also hypothesis-generating. Specifically, these results may represent functional evidence of chronic CO poisoning at the population level, in which chronic, low-level CO exposure induces functional hypoxia and secondary erythrocytosis.~~ Our findings related to CO also align with studies from rural areas of Guatemala and India, which documented evidence of chronic low-level CO exposures linked to household air pollution from cooking with solid biomass.[8,44,45] Of these prior studies, two measured CO exposure through exhaled breath CO levels, [8,45] while one measured

		CO in indoor air using gas-solid chromatography and found concentrations consistently greater than 10 ppm, substantially higher than those in our study.[44] Another study from rural India found high carboxyhemoglobin levels among women regardless of cooking fuel type, suggesting exposure to CO from sources other than cookstoves.[46] CO levels in our study were low at baseline, limiting the potential for further reductions and for stronger exposure-response effects. ~~Our results indicate a need for further investigation to understand the potential prevalence of chronic CO poisoning from cooking and other sources.~~
Some additional comments for the authors to consider:<br><br>Is it possible that the exposure-response CO linear effect is heavily driven by a strong effect in the highest exposed? The effect estimates by exposure quartile do not suggest a monotonic dose response relations, but the effects are large, in relative terms, for Q4 (Table S18). This reviewer is uncertain what the next analysis step might be to further explore this question – a threshold effect? Is it possible that further evaluation of a threshold could reveal more clinically-relevant findings among the highest exposure group?	**Response:** Thank you for this question. We acknowledge the reviewer's point about the results for the highest quartile in the combined analysis shown in Table S18. At the same time, we note that the estimates in the adjusted analyses presented at the bottom of Table S18 are not statistically significant, and categorical associations varied quite a bit by country (Tables S19-S22). Therefore, we do not feel that the data provide evidence of a strong non-monotonic relationship.	(No changes made)
**Reviewer #2 (Remarks to the Author):**		
This is an interesting study.	**Response:** Thank you for the positive assessment of our work and for the specific suggestions, which we have addressed below.	
Methods<br>Lines 142-143. "We sought to randomly assign 1600	**Response:** We have added text to explain that the sample size was determined based	We enrolled 800 women per country and individually randomized them to

participants to the intervention arm and 1600 to the control arm.”. How was the sample size determined?	on the primary outcomes of the trial. This sentence cites the study design paper, which also provides more details on sample size.	intervention or control groups, with intervention households receiving an LPG stove, continuous free fuel delivery, and behavioral reinforcements for 18 months.[16] Women and their infants were followed through the first year of the infant’s life. The sample size was determined based on the p~~P~~rimary outcomes of the trial, which were low birth weight, severe pneumonia, and stunting in infants, along with high blood pressure in non-pregnant adult women residing in the household.[12]
In the statistical analysis section, it is mentioned that “We conducted an intention-to-treat (ITT) analysis to assess the effect of the LPG cookstove and fuel intervention on hemoglobin in pregnant women using a mixed-effects model that takes into account the non-independence of the hemoglobin concentration at each time point.”. However, the time variable was not included in the analysis shown in Table 3. Please clarify.	**Response:** We have deleted the latter part of this sentence, as we recognize that it may have caused confusion. The next sentence, together with the regression equation (now moved to Supplementary Material, Extended Methods), provide more specific information on how time was modeled. As shown in the equation, the ITT analysis did *not* include a fixed effect for time (visit number) in the model presented in Table 3. This was because our primary goal was to estimate the overall effect of the LPG stove and fuel intervention on hemoglobin concentrations across pregnancy, rather than to assess time-specific effects.	We conducted an intention-to-treat (ITT) analysis to assess the effect of the LPG cookstove and fuel intervention on hemoglobin in pregnant women using a mixed-effects model~~ that takes into account the non-independence of the hemoglobin concentration at each time point~~. The two repeat post-intervention hemoglobin measurements were regressed on study arm, with an indicator variable for the ten randomization strata within which the randomization took place (as described above) and a random intercept for each individual, controlling for baseline/pre-intervention hemoglobin levels.
Lines 258-260. “we included variables in the models if they were hypothesized to be predictors of the outcome to decrease the amount of unexplained variability in the data and increase power to estimate	**Response:** We have slightly revised this sentence and added a citation. We did not assess the amount of unexplained variability that would be decreased (or the amount of power that would be increased); we simply	In addition to confounders, we included variables in the models if they were hypothesized to be predictors of the outcome, as this is known to decrease the amount of unexplained variability in the

an association.". It's not clear how the amount of unexplained variability and power were assessed.	included these variables as a standard practice.	data and increase power to estimate an association.[28]
Lines 266-267. "we first fit linear models with different exposure forms (i.e., linear and log-linear)". Please explain why log-linear was used.	**Response:** Thank you for this question. We have added a sentence to this paragraph to explain the use of log-linear models. While normality of exposure itself is not a model requirement, log transformation is a standard practice within air pollution modeling to reflect biological plausibility and improve interpretability and comparability of results with other studies.	Log-linear models are commonly used in air pollution epidemiology to better capture relative exposure–response relationships, improve model fit, and reduce the influence of extreme exposure values.
Line 276. "All analyses were performed using R version 4.2.2". Please provide all packages used in the analysis.	**Response:** We have added a sentence to specify the R package we used for the analysis.	For statistical modeling, we used the R package ImerTest.
Results<br>Table 1. What dose "*" mean?	**Response:** Thank you for catching that the footnote was inadvertently deleted from the table. The footnote has been reinserted.	(See Table 1)<br>** Significantly different at α = 0.05, based on results of t test (continuous variables) or χ2 test (categorical variables) for the difference between intervention and control groups.*
Table 3. Suggest changing a mixed-effect model as a linear mixed-effect model. Noted that in the results of "0.074 g/dL (95% CI: 0.002, 0.145)", it looks that the 95%CI is very wide, which may indicate the uncertainty in the estimate. This should be discussed in the discussion section.	**Response:** Thank you for this suggestion. Rather than changing the title of Table 3, we have added a footnote to the table to clarify this point without making the title overly long and cumbersome.<br><br>With respect to the reviewer's second point, the effect size is relatively small, which makes the confidence interval appear wide, even though the analysis supports statistically significantly higher hemoglobin levels in the intervention group compared to the control. We've added this point to the limitations paragraph within the Discussion section.	See Table 3 footnote:<br>*Linear mixed effect model was used for continuous hemoglobin outcome, and logistic mixed effect model was used for anemia outcome*<br><br>Our main ITT result indicating an effect size of 0.074 g/dL had a 95% confidence interval of 0.002 to 0.145 g/dL, suggesting some uncertainty in the estimate.

Table 4. Noted that the linear or log linear was used. Suggest presenting the backtransformed values and backtransformed 95% CI if log-transformation was used. In addition, how were the missing values handled in the analysis?	**Response:** Thanks for your suggestion regarding the back-transformation of results from log-linear models. After careful consideration, we have decided to add the effect estimates scaled to an IQR increase in exposure, in addition to per unit increase in main text. This addition gives us a more direct comparison as an IQR increase in the unlogged and logged exposure are equivalent. However, we have kept the exposure-response analysis results in Table 4 as per unit increase to facilitate a more meaningful comparison with broader literature in the household air pollution field.<br><br>In addition, we have added a sentence to clarify the handling of missing data. The number of participants with valid exposure and Hb measurements is reported in Table S1 and Figure 1, respectively.	When scaled to the IQR increase in CO exposure, this corresponds to an estimated increase of 0.027 g/dL (95% CI: 0.014, 0.042) g/dL.<br><br>Trial-wide, 1-log-μg/m^3 increase in $PM_{2.5}$ and BC exposures was associated with -0.001 (95% CI: (-0.032, 0.030) g/dL and 0.015 (95% CI: (-0.018, 0.049) g/dL change in hemoglobin concentration, respectively. When scaled to IQR increase in log-transformed exposures, these corresponds to changes of -0.001 g/dL (95% CI: -0.048, 0.045) for $PM_{2.5}$ and 0.022 g/dL (95% CI: -0.027, 0.073) for BC.<br><br>Missing or invalid exposure and Hb measurements were dropped from the model.
**Reviewer #3 (Remarks to the Author):**		
This is an important study of secondary analyses from the large multi-country Household Air Pollution Intervention Network (HAPIN) study. The main randomized controlled trial (RCT) compared liquefied petroleum gas (LPG) fuel and cookstove use to ongoing use of biomass fuels and traditional cookstoves by pregnant women, and multiple primary and secondary results have been published.	**Response:** Thank you for your positive assessment of the importance of our work and for your suggestions, which we have addressed below.	
The general lack of impact of the intervention on anemia rates but positive correlation with hemoglobin concentrations are important findings, as are the findings of an association of CO with a small increase in hemoglobin concentrations. I was surprised to see no	**Response:** Unfortunately, while the HAPIN surveys included questions about dietary diversity, they did not include any questions about consumption of IFA or other supplements. We have added text to	For example, HAPIN did not collect data on consumption of IFA supplements, which could have influenced hemoglobin concentration. However, due to the RCT study design, we anticipate that any

mention of standard of care and iron (of iron folate; IFA) intake in the study population. One assumes that barring severe anemia cases which were excluded, that study pregnant women continued to receive standard antenatal care including IFA. What was the intake in both groups? Was any difference noted by compliance? Did these women receive any other supplements especially as many had a BMI<18.5?	acknowledge this omission as a limitation, while also noting that the random assignment of participants should have led to the balanced distribution of IFA supplement consumption between study arms.	unmeasured covariates would be balanced between intervention and control arms.
Although most women were recruited well after the first trimester (mean gestational age around 15 weeks), were any differences found by length of exposure (those recruited before 14-16 weeks gestation versus late presenters)?	**Response:** Great question. We've conducted this analysis and added text to the Methods and Results sections accordingly. We observed a slightly stronger effect of the intervention on adjusted hemoglobin among participants with gestational age < 15 weeks (estimate: 0.099; 95% CI: -0.007, 0.205), compared to those with gestational age at baseline >= 15 weeks (estimate: 0.044; 95% CI: -0.051, 0.139). However, neither reached statistical significance.	Second, we evaluated the effect of the intervention according to gestational age at study enrollment, with early enrollment being defined as baseline data collection occurring before 15 weeks gestation. In this second analysis, the early and late enrollment groups were each compared with the control group.<br><br>In the exploratory analysis based on timing of study enrollment, hemoglobin in the early enrollment intervention group was 0.099 g/dL (95% CI: -0.007, 0.205) higher than that in the control group, while hemoglobin in the late enrollment intervention group was 0.044 g/dL (95% CI: -0.051, 0.139) higher than that in the control group. However, these results did not reach statistical significance at $p<0.05$.
It would have been useful to show maternal morbidities and birth outcomes by anemia subgroups (moderate, mild or non-anemic women).	**Response:** Thank you very much for this thoughtful suggestion. We agree that these are important research questions to explore. However, the focus of the current analysis is to assess (1) the effect of the HAPIN intervention on hemoglobin concentrations; and (2) the associations between personal	(No changes made)

	exposures to PM2.5/BC/CO and hemoglobin levels, using both continuous hemoglobin values and anemia status as outcomes. Exploring the relationship between anemia and maternal or birth outcomes falls outside the scope of this analysis but is an important area for future research. We appreciate the suggestion and hope to investigate these associations in future analyses.	
**Reviewer #4 (Remarks to the Author):**		
Abstract:<br>1, any clinical consideration for the three timepoints?	**Response:** We have added a sentence to clarify that the time points were selected based on feasibility, not based on clinical considerations.	These time points were selected based on logistical feasibility for the field teams.
2, please check the two values (0.074 g/dL, 95% CI: 0.002, 0.145) vs 0.015 g/dL (95% CI: 0.002, 0.145).	**Response:** We have corrected the typo.	In exposure-response analyses, each 1ppm increase in CO exposure is associated with a 0.015 g/dL (95% CI: 0.008~~2~~, 0.023~~145~~) increase in hemoglobin.
3, Since the CO is part of the air quality, it is very hard to have an intervention aiming on this.	**Response:** Due to word limits, we are not able to add text to the Abstract to address this point. However, we have added a sentence to the Methods section to clarify that the intervention did, indeed, aim to reduce CO levels through the introduction of a cleaner cookstove.	These three pollutants were selected because they are known to be products of incomplete household fuel combustion and because of their associations with adverse health outcomes.[14]
4, multiplicity would be expected after multiply comparisons. Chace findings would not excluded, particularly for CO.	**Response:** Thank you for raising this important point regarding the potential for multiplicity. While we acknowledge the concern, we would like to clarify that the number of comparisons in our manuscript is relatively modest. Specifically, we report approximately 10 ITT results (including country-specific analyses) and 15 exposure–response estimates. These comparisons test	A p-value of <0.05 was considered statistically significant. All tests were two-tailed and, in line with published guidance, we did not adjust for multiple comparisons.[30,31]

	different hypotheses, stratified by different subgroups (trial-wide vs. country-specific) or just finding the best fitting form of exposure, rather than representing a large number of parallel tests of the same association. As such, we did not consider adjustments for multiple comparisons to be necessary in this context. Moreover, even if one were to group all 25 estimates together, the expected number of chance findings at a conventional 5% alpha level would be low. However, we are not interpreting the 25 estimates collectively, but rather as separate research objectives. Finally, we interpret our findings with appropriate caution and do not make clinical recommendations based on our results. We have added a sentence to clarify our approach in the Methods section.	
5, I would recommend stating that further studies are needed to validate this finings rather than focusing on the interven on CO.	**Response:** We have revised the final sentence of the Abstract as suggested.	~~Our finding of positive associations between hemoglobin concentration and CO exposure may represent functional evidence of chronic, population-level CO poisoning~~ Further research may be needed to examine the biological mechanisms underlying our findings.
Introduction<br>Looks fines		
Method.<br>1,This analysis is based on a published study with primary outcomes on low birth weight, severe pneumonia, and stunting in infants, along with high blood pressure in non-pregnant adult women residing in the household. The outcome chosen in this study is a secondary outcome. proper mutilplties needs to be	**Response:** As noted above, we acknowledge the reviewer's concern and also note that many viewpoints exist on multiplicity issues. We have added a sentence and a citation to clarify our approach.	A p-value of <0.05 was considered statistically significant. All tests were two-tailed and, in line with published guidance, we did not adjust for multiple comparisons.[30,31]

took in account. Otherwise, clearly statement about the nature of exploratory analysis is needed to remind the readers.	We also have included text elsewhere in the Methods and Results sections that some of our analyses are exploratory.	We conducted two ~~In an~~additional exploratory analyse~~is~~s.
2, "The HAPIN Data Management Core generated randomization lists based on block randomization using randomly selected block sizes, then prepared individual sealed envelopes containing trial group allocation, which were shipped to each study site and selected by participants" what do you mean selected by participants???	**Response:** We have added a sentence to clarify.	The HAPIN Data Management Core generated randomization lists based on block randomization using randomly selected block sizes, then prepared individual sealed envelopes containing trial group allocation, which were shipped to each study site. Field teams then followed a detailed protocol in which each household was presented with a set of six sealed, sequenced envelopes and asked to select one, which contained their study arm assignment ~~and selected by participants~~.
3, is there analysis plan for this analysis (not this trial)? ITT defined in this analysis did not follow the standard. Why do smokers exclude?	**Response:** We have revised the text to clarify: (1) that the analysis plan mentioned in the text is for this analysis, not for the trial; and (2) that smokers were excluded because current smoking was an exclusion criterion for the trial (as described in the "Study design and participants" sub-section of the Methods) but, in checking the data, we identified 6 people who reported smoking.	Data presented in this manuscript were analyzed in accordance with an analysis plan that was pre-specified and approved prior to securing data access. Participants were excluded from the statistical analysis if they reported any current smoking, in line with trial exclusion criteria.
4, there is no need to describe the formulations with decription is the main text. I would suggest moving this in the appendix or refer to the SAP.	**Response:** We have moved the formulas to the Supplemental Materials, as suggested.	(See Methods and Supplemental Materials)
5, Please define the minimal clinical relevant margin in the method.	**Response:** We did not pre-specify a minimal clinically relevant change. Rather, we simply aimed to evaluate whether the intervention had any impact on hemoglobin concentrations. However, in response to other comments from the reviewer(s), we	The effect size, while statistically significant, was small and likely not clinically meaningful at the individual level.

	have added a paragraph on the clinical relevance of our results in the Discussion section.	
Result:<br>1, a lot of P values in this section without alpha adjustment.	**Response:** As noted above, we acknowledge the reviewer's concern and also note that many viewpoints exist on adjustment for multiple comparisons. We have added a sentence and a citation to clarify our approach.	A p-value of <0.05 was considered statistically significant. All tests were two-tailed and, in line with published guidance, we did not adjust for multiple comparisons.[30,31]
2, any justification for the exclusion of baseline hemoglobin concentrations <7.0 g/dL at baseline?	**Response:** Yes, this is explained in the Methods section.	(No change; below are lines 200-202 from the original submission)<br>Participants with a hemoglobin concentration <7.0 g/dL at baseline were referred for additional management of their anemia. These participants were also dropped from our analysis as we anticipated that their anemia management would affect our outcome of interest.
3, what is the definition of anemia?? the proportion of anemia differ so significantly? Any reason? What is the implication of this group unbalance.	**Response:** We provide the definition of anemia in the Introduction and again in the Methods. As shown in Table 1, the prevalence of anemia at baseline was very similar between the control (32.9%) and intervention (34.4%) groups. Therefore, the groups are well balanced.	(No change; below are lines 71-72 and 225-226 from the original submission)<br>…anemia, defined by the World Health Organization as blood concentrations of hemoglobin less than 12 g/dL (or less than 11 g/dL during pregnancy).<br><br>We created a binary variable for anemia using the WHO cut-off for pregnant women, in which anemia is defined as an adjusted hemoglobin concentration of less than 11 g/dL.
4, please provide the number of subjects for the analysis in Table S23	**Response:** We have added the number of observations to Table S23.	(See Table S23)

Discussion<br>1, Please explain the reasons of having different findings for the anemia and Hb. Currently, the study focus on the HB value. I suspect the outcome reporting bias.	**Response:** We have added text in the first paragraph of the Discussion section to explain these results.	The effect size, while statistically significant, was small and likely not clinically meaningful at the individual level.~~, and t~~ The odds of anemia were not different between women in the intervention group and those in the control group, likely because the small effect on hemoglobin concentration was not sufficient to change anemia status.
2, Positive results related to CO may be a chance finding. How would an intervene would be target the CO	**Response:** As noted above, we have added a sentence to the Methods section to clarify that the intervention targeted CO specifically, with the aim of reducing CO levels through the introduction of a cleaner cookstove.	These three pollutants were selected because they are known to be products of incomplete household fuel combustion and because of their associations with adverse health outcomes.[14]
3, discussion around the clinical relevance and the statisticant significance may be helpful to the readers. The current version mainly place their focus on the statiscant significance.	**Response:** We have revised the first paragraph of the Discussion section and added a new paragraph immediately after that one to address this suggestion. In the new paragraph, we discuss the clinical relevance of our results. We further place our results in context of those from other RCTs that aimed to increase hemoglobin concentrations among pregnant women.	The effect size, while statistically significant, was small and likely not clinically meaningful at the individual level.<br><br>The effect size observed in our study (0.074 g/dL) is small, especially compared to interventions designed to increase iron status through direct approaches for an individual person or patient. For example, the standard of care for pregnant women is daily oral iron and folic acid (IFA) supplementation.[32] A recent systematic review and meta-analysis found that daily oral iron supplementation had a mean effect size on hemoglobin concentration among pregnant women at or near term (≥34 weeks gestation) of 0.953 g/dL (95% CI: 0.699, 1.206).[33] RCTs of other interventions to increase iron intake

		among pregnant women through dietary supplementation have had mixed results: iron-fortified beverages were found to increase Hb concentrations (0.416 g/dL) in Tanzania but not in Vietnam, while daily food supplementation was found to have no effect on Hb concentration in Indonesia.[34-36] Another RCT of iron-fortified food supplementation among pregnant women in Cambodia did not report Hb concentrations but reported a significant protective effect on anemia (Odds Ratio=0.51; 95% CI: 0.34, 0.77).[37] The observed effect size in our trial, while not clinically meaningful alone, is larger than has been observed in some dietary interventions and could complement the standard of care IFA supplementation as part of a larger public health, population-level anemia prevention and control strategy.

Response to reviewers

We thank the reviewers again for their careful review and suggestions, which have strengthened the manuscript. We have responded to the remaining comments below.

Reviewer 3: I think the authors have responded reasonably well to the critique and comments. In terms of the analysis of impact by anemia categories, it is a natural question readers may ask and including a sentence as to why this isn't part of the current analysis, or not feasible, would be important. Providing some of that prevalence data from the baseline would also allow a comparative evaluation of the population in reference to other geographies.

Response: As requested, descriptive statistics of anemia prevalence by anemia category (severe, moderate, and mild) from baseline has been added to Table 1. We have also added a sentence to the Discussion section to suggest that while an examination of impact on maternal morbidity by anemia categories was outside the scope of our current analysis, this would be an appropriate future research direction.

Reviewer 4: The authors have satisfactorily addressed my previous concerns. I have one further comment regarding the clinical relevance of the findings in light of the small effect size. I recommend that this point be explicitly highlighted in the Discussion section to ensure readers are aware of its implications.

Response: As suggested, we explicitly highlight this point in the first paragraph of the Discussion, where the third sentence is: “The effect size, while statistically significant, was small and likely not clinically meaningful at the individual level.”
